# Increased breadth of HIV-1 neutralization achieved by diverse antibody clones each with limited neutralization breadth

**DOI:** 10.1371/journal.pone.0209437

**Published:** 2018-12-19

**Authors:** Valentine U. Chukwuma, Nurgun Kose, D. Noah Sather, Gopal Sapparapu, Rachel Falk, Hannah King, Vidisha Singh, Rebecca Lampley, Delphine C. Malherbe, Noah T. Ditto, Jonathan T. Sullivan, Trevor Barnes, Benjamin J. Doranz, Celia C. Labranche, David C. Montefiori, Spyros A. Kalams, Nancy L. Haigwood, James E. Crowe

**Affiliations:** 1 Department of Pathology, Microbiology and Immunology, Vanderbilt University Medical Center, Nashville, Tennessee, United States of America; 2 Vanderbilt Vaccine Center, Vanderbilt University Medical Center, Vanderbilt University, Nashville, Tennessee, United States of America; 3 Center for Infectious Disease Research, Seattle, Washington, United States of America; 4 Department of Pediatrics, Vanderbilt University Medical Center, Nashville, Tennessee, United States of America; 5 Oregon National Primate Research Center, Oregon Health & Science University, Beaverton, Oregon, United States of America; 6 Carterra Inc., Salt Lake City, Utah, United States of America; 7 Integral Molecular, Inc., Philadelphia, Pennsylvania, United States of America; 8 Division of Surgical Sciences, Duke University, Durham, North Carolina, United States of America; 9 Department of Medicine, Vanderbilt University Medical Center, Nashville, Tennessee, United States of America; New York State Department of Health, UNITED STATES

## Abstract

Broadly neutralizing antibodies (bNAbs) are rarely elicited by current human immunodeficiency virus type 1 (HIV-1) vaccine designs, but the presence of bNAbs in naturally infected individuals may be associated with high plasma viral loads, suggesting that the magnitude, duration, and diversity of viral exposure may contribute to the development of bNAbs. Here, we report the isolation and characterization of a panel of human monoclonal antibodies (mAbs) from two subjects who developed broadly neutralizing autologous antibody responses during HIV-1 infection. In both subjects, we identified collections of mAbs that exhibited specificity only to a few autologous envelopes (Envs), with some mAbs exhibiting specificity only to a subset of Envs within the quasispecies of a particular sample at one time point. Neutralizing antibodies (NAbs) isolated from these subjects mapped mostly to epitopes in the Env V3 loop region and the CD4 binding site. None of the individual neutralizing mAbs recovered exhibited the cumulative breadth of neutralization present in the serum of the subjects. Surprisingly, however, the activity of polyclonal mixtures comprising individual mAbs that each possessed limited neutralizing activity, could achieve increased breadth of neutralizing activity against autologous isolates. While a single broadly neutralizing antibody targeting one epitope can mediate neutralization breadth, the findings presented here suggest that a cooperative polyclonal process mediated by diverse antibodies with more limited breadth targeting multiple epitopes also can achieve neutralization breadth against HIV-1.

## Introduction

The primary goal of most current HIV-1 research efforts has been aimed at isolating and characterizing cross-reactive and potent neutralizing antibodies (bNAbs) [1] that can limit or prevent infection in animal models and potentially humans [2]. This work has led to an increased focus on understanding how these bNAbs are elicited [3], and on elucidating the factors associated with the development of bNAbs during the course of natural HIV-1 infection [4].

A major impediment to the successful development of a protective HIV-1 vaccine is the enormous antigenic variability of the HIV-1 Env glycoprotein associated with the diverse viral quasispecies that develop during HIV-1 infection [5]. A successful vaccine should induce broadly neutralizing antibodies (bNAbs) that can recognize a very diverse array of viral isolates, including antigenically distinct viruses that occur in different geographic locations [6]. Over the last three decades, several Env-based immunogens have been tested as vaccine candidates, with limited success in conferring protection against HIV-1 [7].

Although many HIV-1 infected individuals generate NAbs within the first year or two of infection, such antibodies early in the course of infection usually possess limited neutralization breadth [8]. The typical NAbs induced early in infection neutralize previous autologous viral isolates but show limited neutralization breadth when tested against heterologous viral isolates [9]. However, depending on testing methods, bNAbs have been shown to arise within the first three years of HIV-1 infection in about 10 to 30% of HIV-1-infected individuals [10,11,12]. Similar antibodies have rarely been elicited by current vaccine design efforts, but studies have shown that the presence of bNAbs in naturally infected individuals may be associated with high plasma viral loads [13,14]. These observations suggest that in some chronically infected subjects, the magnitude, duration, and diversity of viral exposure may contribute to the development of bNAbs [15].

Over the last few decades, a number of HIV-1 bNAbs have been isolated from chronically infected subjects, and the specificity of these mAbs has been mapped to diverse antigenic regions on the HIV-1 Env [16,17,18]. One class of bNAbs targets the membrane-proximal external region (MPER) of gp41 [19,20,21], with antibody clones that often exhibit a demonstrable level of autoreactivity [22]. A number of bNAbs target the CD4 binding site (CD4bs) of the HIV-1 Env, but overall only a few HIV-infected individuals make those [23,24,25]. Members of a potent class of CD4bs bNAbs are encoded by the IGHV1-2*02 human V_H_ allele, and studies on germline-targeting immunogens have suggested that bNAb VRC01-class precursors occur in 96% of individuals with the right IGHV1-2 allele [26]. Also, many CD4bs antibodies have undergone extensive somatic hypermutation [27]. The induction of such bNAbs by vaccination will not only prove challenging, but also likely would require a vaccine regimen that includes multiple boosts to drive the somatic hypermutation process to an extent seen only in chronic natural infection [28]. One group of bNAbs targets the variable loops on the HIV-1 Env, and many of these antibodies have been reported to have very long heavy chain complementarity determining region 3 (HCDR3) loops [29]. An important group of bNAbs targeting high mannose glycans on the Env surface [30,31] also has been reported. BNAbs targeting a novel epitope at the gp120-gp41 interface have been identified [32].

The genetic and structural features of some of these previously isolated bNAbs provide insights into the molecular basis for immunogenicity, which informs the design of vaccine strategies that can be used to elicit bNAbs. Studies have shown that affinity maturation plays an important role in the development of most bNAbs [33]. An investigation into the co-evolution of a founder virus and a neutralizing antibody lineage revealed that incremental somatic mutations during the affinity maturation process led to the progressive development of breadth and activity in a bNAb lineage [34]. Other studies have reported that more than one clonal lineage pathway can lead to the development of bNAbs [35]. However, there is still a limited understanding of the natural evolutionary mechanisms that lead to the development of breadth in the polyclonal antibody repertoire of HIV-1 infected subjects. Most of our understanding of the activity of bNAbs from prior studies comes from isolation of individual mAbs from many different patients, rather than from characterization of the repertoire of antibodies in a single patient. Studies have shown that the neutralizing antibody response in an individual with triple HIV-1 infection is directed against the first infecting subtype [36]. An in-depth evaluation of the neutralizing antibody response to viral evolution within individual infected subjects provides insights into the pathways that can lead to the acquisition of a broadly neutralizing antibody response in HIV-1 infected individuals. Longitudinal sampling from time to infection to the development of neutralization breadth reveals that a V_H_1-46 derived antibody lineage directed at the CD4 binding site, matured to acquire neutralization breadth through epitope focusing and extraordinary levels of somatic hypermutation [37].

Here, we report the isolation and characterization of panels of HIV-1 mAbs over time from two subjects with chronic infection who developed serologic evidence of neutralizing breadth. Previous serological studies on these subjects reported the development of neutralization breadth against autologous viruses isolated over the course of multiple years after infection and against a representative panel of HIV-1 field strains [11,38,39]. Both subjects exhibited evidence for moderately broad serum neutralizing antibody responses against autologous viruses that focused on distinct major antigenic sites on Env. The serum neutralizing activity from one subject (designated subject VC10014) was mapped to an antigenic site that overlaps the CD4 receptor-binding site, while the serum neutralizing activity from the second subject (designated subject VC20013) was mapped to the MPER region of gp41 [38]. It was not clear from those studies whether the breadth of neutralization activity in the serum stemmed from individual clones with unusual breadth or from an ensemble of antibodies with limited breadth that comprised a polyclonal response with broad composite activity.

In this study, we isolated panels of mAbs from these two subjects and characterized the binding and neutralization phenotypes for autologous and heterologous viruses. A subset of mAbs from each of the subjects exhibited potent neutralization against autologous isolates, and some heterologous Tier 1 clade B HIV-1 isolates, but overall, showed very limited breadth against heterologous isolates. This group of mAbs from both subjects mapped predominantly to the CD4bs and V3 loop region of the HIV-1 Env. Most of the mAbs isolated recognized only specific isolates, or subsets of isolates. Interestingly, we did not identify any one clone that exhibited the full breadth of autologous or heterologous neutralizing activity seen in late time-point serum samples. However, combinations of mAbs recognized diverse subsets of autologous viruses and achieved increased breadth compared to individual mAbs. The findings from these studies suggest that increases in autologous neutralization breadth can be achieved by accumulating diverse antibody clones that mediate breadth by a cooperative polyclonal process.

## Materials and methods

### Ethics statement

The study was approved by the Institutional Review Board at the Vanderbilt University School of Medicine (Vanderbilt University Medical Center, Nashville, TN, USA), and the study participants provided written informed consent prior to participating in this study.

### Human subjects and peripheral blood cell isolation

The subjects enrolled in the Vanderbilt/CFAR cohort (indicated by the prefix VC) were described previously [11,38]. Subjects VC10014 and VC20013 described in this study were volunteers with known times of seroconversion, who were not on antiretroviral therapy (ART) and with times since infection of less than 1 year, who developed moderate bNAbs in less than three years of infection. Twelve blood samples from subject VC10014, and nine blood samples from subject VC20013 were collected at different time points following infection and analyzed. Peripheral blood mononuclear cells (PBMCs) were isolated by density gradient separation on Ficoll. The cells were cryopreserved and stored on liquid nitrogen until study.

### EBV-transformation of B cells

Previously cryopreserved PBMCs were transformed using Epstein-Barr virus (EBV), as previously described [40]. Briefly, samples were thawed rapidly in a 37°C water bath, washed once in 10 mL of Medium A (Stemcell Technologies), counted and viability assessed with trypan blue staining (Gibco). An aliquot of 4 million viable cells was mixed with Medium A, CpG, cyclosporine, the apoptosis inhibitor Chk2i, and clarified supernatant from cultures of B95.8 cells (ATCC) containing Epstein-Barr virus (EBV). The mixture then was plated into 384-well plates (Nunc), and plates were incubated at 37°C in 5% CO_2_ for 10 days. Cells were expanded into a 96-well flat bottom plate in 200 μL of Medium A containing gamma-irradiated healthy donor PBMCs mixed with CpG, and Chk2i. Plates were incubated at 37°C for 4 days prior to screening for HIV-1-reactive B cell lines in an enzyme-linked immunosorbent assay (ELISA) using Env or a TZM-bl-based neutralization assay using pseudoviruses. The plates then were incubated for an additional 3 to 4 days, and cells from wells with supernatants reacting in a HIV-1 Env-specific ELISA or neutralization assay were fused with HMMA2.5 non-secreting myeloma cells to form hybridomas.

### Electrofusion of EBV-transformed B cells with myeloma fusion partner

HIV-1 specific B cells were fused with HMMA2.5 myeloma cells using a previously described protocol [40]. Briefly, HMMA2.5 cells were counted and suspended at 10 million cells/mL in a microcentrifuge tube in 1 mL of warmed cytofusion medium. HIV-1-reactive EBV-transformed B cell wells were pipetted gently into microcentrifuge tubes containing 1 mL of warmed cytofusion medium. After four washes, the HMMA2.5 cell suspension was mixed with each tube of EBV-transformed B cells, and the mixture was pipetted into cytofusion cuvettes (BTX) and placed in a modified cytofusion device. After fusion, the cuvettes were incubated at 37°C for 30 min and the contents of each cuvette was added to 20 mL of hypoxanthine-aminopterin-thymidine (HAT) medium containing ouabain (Sigma), Medium A, Medium E (Stemcell Technologies) and HAT (Sigma). Fusion products then were plated into 384-well plates, incubated at 37°C for 21 days, and then hybridomas screened for antibody production by ELISA using recombinant Env protein.

### MAb production and purification

MAbs were isolated using a previously described protocol [40]. Briefly, hybridomas producing HIV-specific antibodies were cloned biologically by two rounds of limiting dilution plating, followed by one round of single-cell sorting using flow cytometry to achieve clonality. Each hybridoma was expanded in 75-cm^2^ flasks (Corning) in Medium E until 50% confluent. For antibody expression, the hybridomas in a 75-cm^2^ flasks were collected with a cell scraper, washed in serum-free medium (Gibco) and split equally among four 225-cm^2^ flasks (Corning) containing 250 mL of serum-free medium. The flasks then were incubated for 21 days, and the medium was clarified by centrifugation and sterile filtered (0.2-μm pore size). The antibodies were purified by chromatography using protein G columns (GE Life Sciences).

### Screening enzyme-linked immunosorbent assay

HIV-1 Env gp140 protein prepared in carbonate binding buffer was used to coat ELISA plates (Nunc) and incubated at 4°C overnight. The plates were blocked by incubation at room temperature for 1 h with 50 μL of 2% blocking solution/well. Blocking solution consisted of 20 g of powdered milk, 20 mL of goat serum, 100 mL of 10X phosphate-buffered saline (PBS), and 0.5 mL of Tween (Sigma) mixed to a 1 L final volume with distilled water. Plates were washed four times with PBS-T, and 25 μL of supernatant was transferred from one well of a 384-well plate containing EBV-transformed B cell lines, using a ViaFlow 384 device (Integra) into 5 μL of blocking solution/well. The plates were incubated at room temperature for 1 h, followed by four additional washes with PBS-T. Goat anti-human IgG-HRP (SouthernBiotech) was applied at a 1:3,000 dilution in blocking solution using 25 μL/well, and the plates were incubated at room temperature for 1 h. After four washes with PBS-T, 25 μL of 1-Step Ultra TMB-ELISA solution (Pierce) was added, and the reaction was stopped with 25 μL 1N HCl. Absorption at 450 nm was read on a Molecular Devices plate reader.

### Production of pseudoviruses

HIV pseudoviruses were produced using the Env protein sequences from circulating viruses isolated from subjects VC10014 and VC20013 [38], a standard reference panel of subtype B HIV-1 Env clones, and a standard reference panel of subtype C HIV-1 Env clones (NIH AIDS Reagent Program) using a previously described protocol [41]. Briefly, 293T/17 cells were plated for 24 h at a density of 5 x 10^5^ cells/mL. 8 μg of the pNL4-3.Rev-.Env-.Luc+ HIV backbone and 4 μg of *env* plasmid DNA were mixed in a complex of Opti-MEM I medium (Life Technologies) and Fugene-6 transfection reagent (Promega). The mixtures were incubated for 20 min to precomplex, and then added to the 293T/17 cells. After 6 h, the medium was removed and fresh growth medium [Dulbecco modified Eagle medium (Life Technologies) supplemented with gentamicin, HEPES and 10% fetal bovine serum (Gibco)] was added to the culture. After incubation at 37°C for 48 h, the supernatant was harvested, clarified by centrifugation, and stored at -80°C.

### Neutralization assays

The neutralizing activities of antibodies in supernatants from EBV-transformed B cell lines or in suspensions of purified mAbs were determined using the TZM-bl-based neutralization assay [42,43]. Briefly, antibodies were pre-incubated with pseudovirus for 60 min at 37°C. The antibody/pseudovirus mixture was added to TZM-bl cells (10,000 cells per well in a 96-well plate) for 48 h at 37°C. 100 μL of the supernatant was removed and 100 μL of Bright-Glo Luciferase Assay Substrate (Promega) was added to each well. Plates were incubated for 2 min at room temperature and 150 μL of the lysate was transferred to microtiter plates. The cell-associated luciferase activity for each well was determined on a Modulus microplate luminometer (Turner Biosystems). Percent neutralization was calculated for each antibody as the percent inhibition of viral entry by the antibody compared to the absence of antibody. For each mAb /pseudovirus combination tested, a neutralization curve (percent neutralization versus antibody dilution) was generated using GraphPad Prism version 4.0 (GraphPad Software), and the antibody dilution at which 50% neutralization was recorded (IC_50_) was determined by transforming the data to a log_10_ scale and fitted with sigmoidal dose response curves.

### Mapping linear epitopes using overlapping peptides

The HIV-1 consensus group M Env peptide set (NIH AIDS Reagent Program; catalog number 9487) was used to map linear epitopes of mAbs. For detection of antibody binding, peptides were prepared in carbonate binding buffer, and used to coat ELISA plates (Nunc). After overnight incubation at 4°C, plates were washed six times with PBS-T (PBS with 0.05% Tween-20) and blocked for 2 h at room temperature with 50 μL per well of blocking solution. Plates were washed six times with PBS-T, and 25 μL of mAbs at a concentration of 5 μg/mL was added to each well. Plates were incubated for 2 h at room temperature and washed six times with PBS-T. Goat anti-human IgG-HRP (SouthernBiotech) was added at a 1:3,000 dilution in blocking solution using 25 μL/well, and the plates were incubated for 1 h at room temperature. After four washes with PBS-T, 1-Step Ultra TMB-ELISA solution (Pierce) was added at 25 μL/well, and the reaction was stopped with 25 μL 1N HCl. Absorption at 450 nm was read on a Molecular Devices plate reader.

### Epitope binning using surface plasmon resonance

The recombinant gp140 Env proteins used in these assays were produced as previously described [44]. Antibodies were prepared to 10 μg/mL in 10 mM sodium acetate pH 4.5 and arrayed on a carboxymethylated dextran sensor surface activated with a mixture of 1-ethyl-3-(3-dimethylaminopropyl) carbodiimide hydrochloride (EDC) and N-hydroxysuccinimide (NHS) using a Continuous Flow Microspotter (Wasatch Microfluidics). To assess competitive relationships among mAbs, 75 nM of SF162 gp140 was injected across the mAb array followed by injection of each mAb at 10 μg/mL sequentially using the MX96 SPR imager (IBIS Technologies) in PBS, 0.01% Tween-20 running buffer. Surface mAbs were regenerated using 50 mM glycine pH 2.0. Binding responses were normalized and structured into a hierarchical clustered heat map. Community detection algorithms were applied to derive network plots representing competitive relationships among mAbs as previously described [45].

### Alanine scanning mutagenesis

A shotgun mutagenesis mutation library was created for HIV-1 envelope protein gp160 (strain KNH1144, GenBank #JQ715384), as previously described [46]. Briefly, a parental plasmid expressing codon-optimized gp160 Env was used as a template to make a library of alanine mutations, while alanine residues were mutated to serine. Each mutant was transfected individually into human HEK-293T cells and after a 22 h expression, cells were fixed in 4% (v/v) paraformaldehyde (Electron Microscopy Sciences, Hatfield, PA) in PBS plus calcium and magnesium (PBS++). Purified mAbs were used to stain cells, and mAbs were detected using AlexaFluor488-conjugated secondary Ab (Jackson ImmunoResearch Laboratories, Pike West Grove, PA). Cells were washed 3 times with PBS, and mean cellular fluorescence was detected using the Intellicyt High-Throughput Flow Cytometer (HTFC, Intellicyt, Albuquerque, NM). MAb reactivities against each mutant gp160 clone were calculated relative to wild-type Env reactivity by subtracting the signal from mock-transfected controls and normalizing to the signal from wild-type Env-transfected controls. Residues required for mAb binding were identified as critical to the mAb epitope if reactivity of the test mAb was lost, but reactivity of control Abs was retained. This counter-screen strategy enables the exclusion of gp160 mutants that are locally mis-folded or have an expression defect [47].

## Results

### Human mAbs to HIV Env protein

We isolated a panel of human anti-HIV-1 mAbs from two human subjects, designated VC10014 and VC20013, who had developed broad cross-clade serum neutralizing antibody responses during the course of HIV-1 infection. Details about the donors for the mAbs are shown in (S1 Table). The serologic response of these subjects to HIV-1 infection was described previously [38]. That study showed that sequential plasma samples from both subjects revealed an incremental acquisition of neutralization breadth when tested against autologous viral isolates representing the predominant circulating strains at different time-points during the course of infection. We isolated a total of 90 human mAbs from VC10014 and 56 mAbs from VC20013 using PBMCs obtained from blood samples at four time-points for each donor. PBMCs were obtained specifically 2.84, 3.22, 3.59 or 5.80 years post-infection for subject VC10014 and 2.12, 2.6, 7, or 7.9 years post-infection for subject VC20013 (S1 Table). HIV-1-reactive B cell lines were present in about 15% of total wells tested depending on the efficiency of EBV-transformation, and the HIV-1 neutralization frequency of EBV-transformed B cell lines in both subjects ranged from 0.07–0.11%. HIV-1 reactive B cells were fused with myeloma cells and passed through several rounds of limiting dilution to generate cloned hybridoma cells secreting HIV-1 specific human mAbs.

### Differential binding and neutralization phenotypes of EBV-transformed B cell supernatants

**Fig 1:**
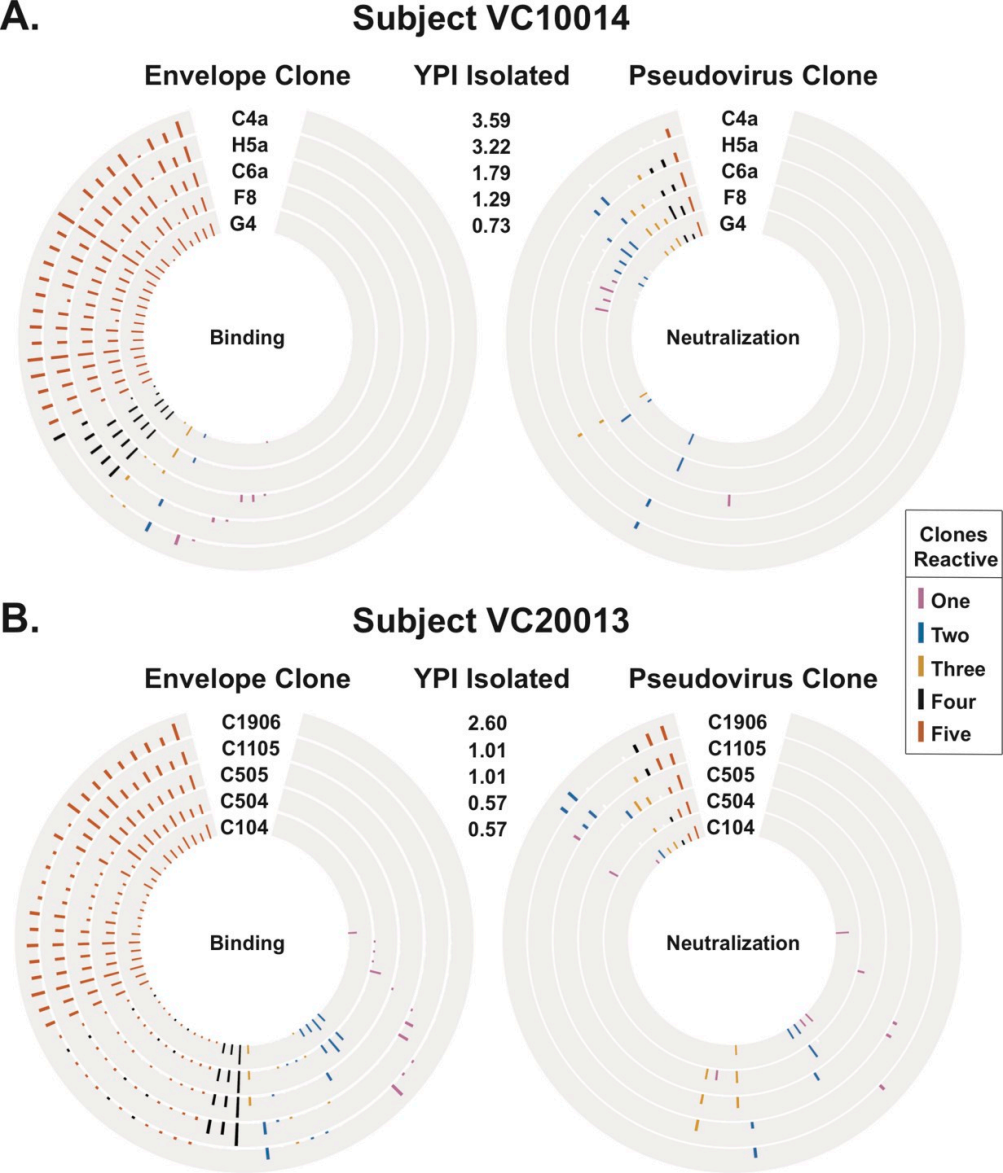
Differential binding and neutralization of EBV-transformed B cell supernates. Circos plot showing binding or neutralization of B cell supernatants as determined by ELISA and neutralization assays. Each colored bar represents positive binding or neutralizing activity for B cell supernatants from a single well of a 384-well plate, with height of bar indicating intensity of binding or neutralization. **(A)** The binding and neutralization of EBV-transformed B cell supernatants from subject VC10014 was determined by ELISA and neutralization assays using Env gp140 proteins and pseudoviruses made from Env sequence of five autologous viruses from 0.73 (G4), 1.29 (F8), 1.79 (C6a), 3.22 (H5a), 3.59 (C4a) YPI. **(B)** The binding and neutralization of EBV-transformed B cell supernatants from subject VC20013 was determined by ELISA and neutralization assays using Env gp140 proteins and pseudoviruses made from Env sequence of five autologous viruses from 0.57 (C104, C504), 1.01 (C505, C1105), 2.60 (C1906) YPI. The number of reactive clones for each supernatant is color-coded as 1(reddish purple), 2 (blue), 3 (orange), 4 (black), or 5 (vermillion).

To determine the specificity of HIV-1 reactive B cell lines, we tested antibodies in the supernatants of EBV-transformed B cells for binding to autologous gp140 proteins (Env) and neutralization of the matching autologous pseudoviruses generated using gp160 obtained from sequencing the Env of five autologous viruses for each subject. The previously published Env names and GenBank accession numbers are listed in S2 Table. For subject VC10014, the Env sequences were from 0.73 (G4), 1.29 (F8), 1.79 (C6a), 3.22 (H5a), or 3.59 (C4a) years post-infection (YPI), and 0.57 (C104, C504), 1.01 (C505, C1105), or 2.60 (C1906) YPI for subject VC20013. In both subjects, we observed that majority of the 384 supernatants tested from uncloned EBV-transformed B lymphoblastic cell lines or BLCLs (one supernate per well of a 384-well plate) did not bind to or neutralize any of the gp140s or pseudoviruses tested (Fig 1). These data indicate that in these subjects, the HIV-1 reactive B cell population during chronic HIV-1 infection was a small subset of the overall repertoire, consistent with previous studies [48,49]. We identified several BLCLs that were reactive to the Envs and pseudoviruses tested. These patterns of reactivity are color-coded as 1 (reddish purple), 2 (blue), 3 (orange), 4 (black), or 5 (vermillion) in Fig 1. We observed that a majority of the HIV-1-reactive BLCLs in both subjects showed binding to all five gp140s, with a smaller subset showing specificity to one, two, three or four of the gp140s tested. One of the major findings from these experiments was the observed differential binding and neutralization of the different BLCLs tested. For subject VC10014, for whom all gp140s were isolated from different YPI, we identified eight BLCLs [reddish purple bars in Fig 1A left panel] with binding specificity to only one Env for gp140s isolated 0.73, 1.79, 3.22 or 3.59 YPI (Fig 1A, left panel). From this group, only one of the BLCLs neutralized the matching pseudovirus (Fig 1A, right panel). Within the subset of BLCLs that showed binding to all five gp140s [vermillion bars in Fig 1A left panel], we observed differential neutralization phenotypes when tested for neutralization of the matching pseudoviruses. In addition to one BLCL that neutralized all of the matching pseudoviruses, we identified BLCLs within this subset that showed binding to all five gp140s, but only neutralized one, two, three or four of the matching pseudoviruses tested (Fig 1A, right panel). For subject VC20013, from whom more than one gp140 was isolated from a particular time-point (0.57 and 1.01 YPI), we identified nine BLCLs [reddish purple bars in Fig 1B left panel] that showed binding only to one of the time-point specific gp140s (Fig 1B, left panel). Several BLCLs from this group also neutralized the matching pseudoviruses (Fig 1B, right panel). The same pattern was observed for seven BLCLs that showed specificity to gp140s from more than one time-point but showed binding to only one of the time-point specific gp140s (Fig 1B, left panel). Within the group of BLCLs from subject VC20013 that showed binding to all five gp140s tested [vermillion bars in Fig 1B left panel], we identified subsets that neutralized one, two, three, four or all of the matching pseudoviruses tested. In this group, we observed several BLCLs with neutralization specificity for one, but not the other pseudovirus from the same time-point. We also identified BLCLs that showed time-point specific neutralization within this population. A few BLCLs [vermillion bars in Fig 1B right panel] neutralized all of the pseudoviruses tested (Fig 1B, right panel). These findings indicate that in the antibody repertoire of HIV-1 infected subjects, individual B cells secrete Abs that exhibit diverse binding and neutralization patterns against several isolates in the quasispecies of circulating autologous viruses.

### Differential antibody binding to HIV-1 Env glycoproteins

**Fig 2:**
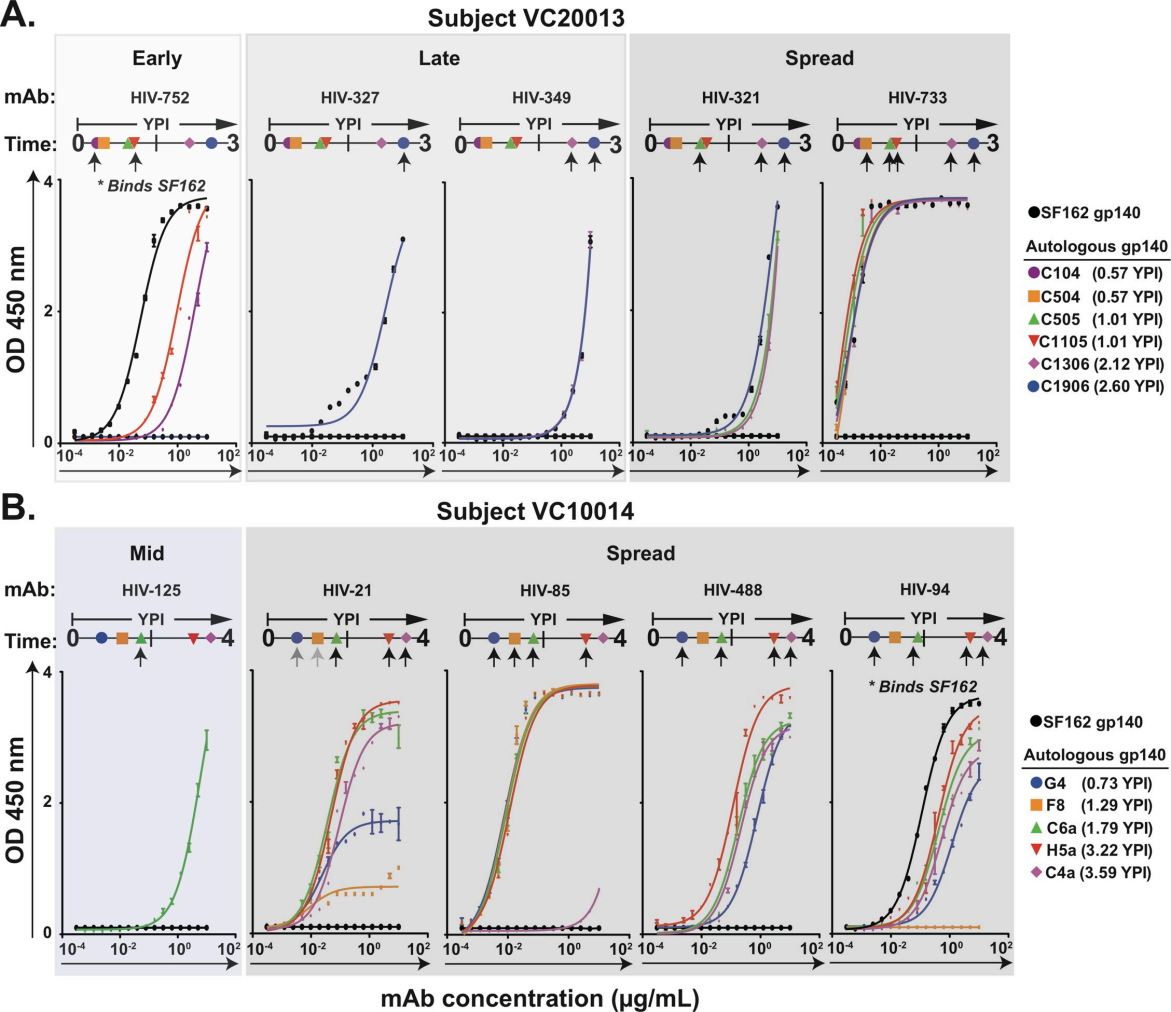
Differential binding of mAbs to Envs from different years post infection. ELISA was used to determine binding. **(A)** The binding of representative mAbs from subject VC20013 was determined by ELISA using Env gp140 proteins made from Env sequence of six autologous viruses from 0.57 (C104, C504), 1.01 (C505, C1105), 2.12 (C1306), 2.60 (C1906) YPI, and heterologous Env SF162. **(B)** The binding of representative mAbs from subject VC10014 was determined by ELISA using Env gp140 proteins made from Env sequence of five autologous viruses from 0.73 (G4), 1.29 (F8), 1.79 (C6a), 3.22 (H5a), 3.59 (C4a) YPI. Dark arrow represents strong binding to indicated Envs, grey arrow represents weak binding to indicated Envs.

In order to characterize more precisely the differential patterns of recognition observed from the EBV-transformed BLCLs, we performed binding assays using the panel of the clonal mAbs isolated from each subject against the corresponding panel of autologous gp140s. We observed differential gp140 binding patterns to viruses from phases designated as early, mid, late or spread (early, mid and late) phases of infection. In subject VC10014, the early phase is represented by mAb binding to gp140s from 0.73 or 1.29 YPI, the mid phase is represented by mAb binding to gp140 from 1.79 YPI, and late phase is represented by mAb binding to gp140s from 3.22 or 3.59 YPI. In subject VC20013, the early phase is represented by mAb binding to gp140s from 0.57 YPI, the mid phase is represented by mAb binding to gp140s from 1.01 YPI, and the late phase is represented by mAb binding to gp140s from 2.12 or 2.60 YPI. In both subjects, the spread phase is represented by differential mAb binding to gp140s across the different time-points. Binding curves for some mAbs with differential binding patterns are shown for subject VC20013 (Fig 2A), and subject VC10014 (Fig 2B). From subject VC20013, mAb HIV-752 isolated at 7 YPI showed early gp140 specificity with binding only to gp140s from 0.57 and 1.01 YPI. Interestingly, within each time-point, mAb HIV-752 showed binding specificity to one, but not to the second, gp140 (Fig 2A, first graph). MAbs HIV-327 isolated at 2.60 YPI and HIV-349 isolated at 2.12 YPI showed late gp140 specificity, with both mAbs binding to the C1906 gp140 from 2.60 YPI. In addition, mAb HIV-349 showed binding to the C1306 gp140 from 2.12 YPI (Fig 2A, third graph). MAbs HIV-321 isolated at 2.60 YPI and HIV-733 isolated at 7.9 YPI showed spread gp140 specificity. MAb HIV-321 showed binding to gp140s from 1.01 YPI (C505), 2.12 YPI (C1306), and 2.60 YPI (C1906). MAb HIV-733 showed binding to all the gp140s tested, except for C104 gp140 from 0.57 YPI (Fig 2A, fifth graph). MAb HIV-752 showed binding to SF162 gp140, but we did not detect binding to SF162 gp140 for the other four mAbs from subject VC20013, shown in Fig 2A. From subject VC10014, mAb HIV-125 isolated at 3.22 YPI showed mid phase binding specificity, with binding only to the C6a gp140 from 1.79 YPI (Fig 2B, first graph). The rest of the mAbs described from subject VC10014 showed a spread phase binding specificity. MAb HIV-21 isolated at 3.86 YPI showed binding to all the gp140s tested. However, we observed reduced maximum binding thresholds to the early gp140s from 0.73 and 1.29 YPI (Fig 2B, second graph). MAb HIV-85 isolated at 3.86 YPI showed binding to all the gp140s tested, with the exception of the C4a gp140 from 3.59 YPI, for which binding was not detected (Fig 2B, third graph). MAbs HIV-488 isolated at 2.84 YPI and HIV-94 isolated at 3.86 YPI both showed binding to all the gp140s tested, with the exception of the F8 gp140 from 1.29 YPI, for which binding was not detected. Interestingly mAb HIV-94 also showed binding to the SF162 gp140 (Fig 2B, fifth graph). All of the other representative mAbs from subject VC10014 did not show binding to SF162 gp140, as shown in Fig 2B. These results from binding studies using mAbs validated our earlier findings from EBV-transformed B cells which showed that individual B cells in HIV-1 infected subjects secrete Abs that differentially recognize various HIV-1 isolates.

### Autologous neutralization of anti-gp140 mAbs

**Fig 3:**
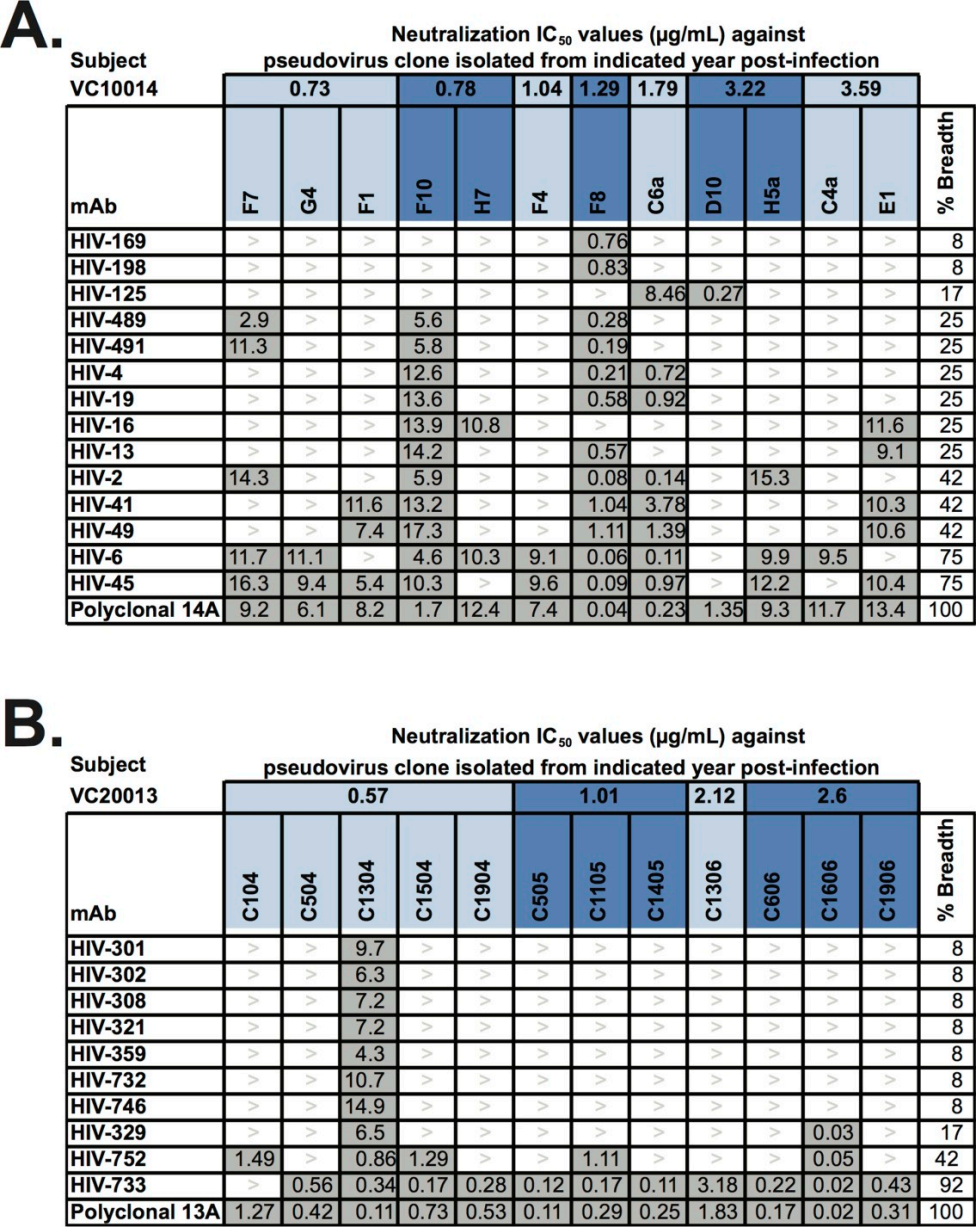
Autologous neutralization breadth of HIV-1 mAbs. The neutralization phenotypes of HIV-1 mAbs from subjects VC10014 and VC20013 were determined using neutralization assays. **(A)** Neutralization phenotype of mAbs and polyclonal mixture from subject VC10014 against autologous pseudoviruses from indicated years post infection. **(B)** Neutralization phenotype of mAbs and polyclonal mixture from subject VC20013 against autologous pseudoviruses from indicated years post-infection. The values reported here shaded in grey are the IC_50_ values of neutralization as antibody concentration in μg/mL. The percent breadth reported is calculated by dividing the number of pseudoviruses for which an IC_50_ was recorded by the total number of pseudoviruses tested and multiplying by 100.

To evaluate the neutralization activity of the panel of mAbs from both subjects, we first performed neutralization assays using pseudoviruses based on the Env sequence of autologous viruses isolated from each subject at different time-points during the course of HIV-1 infection. For subject VC10014, we tested pseudoviruses from 0.73 [two isolates], 0.78 [three isolates], 1.04 [one isolate], 1.29 [one isolate], 1.79 [one isolate], 3.22 [two isolates], 3.59 [one isolate], or 3.86 [one isolate] YPI. We found that 14 out of the 90 mAbs isolated from subject VC10014 exhibited neutralization activity against at least one of the autologous pseudoviruses tested (Fig 3A). The differential binding and neutralization of all the mAbs generated from subject VC10014 is shown in (S4 Table). The genetic and functional features of these mAbs are summarized in (S5 Table). These mAbs exhibited differential patterns of neutralization with neutralization breadth ranging from 8 to 75% of pseudoviruses tested. The neutralizing activity of these mAbs against the panel of autologous pseudoviruses was characterized as predominantly moderately to weakly neutralizing, with most IC_50_ values greater than 5 μg/mL. Some of these mAbs were isolated at 5.80 YPI, and the matching subject VC10014 plasma sample from the same time-point showed neutralizing activity when tested against 100% of this panel of autologous pseudoviruses. Therefore, we tested a polyclonal mixture [polyclonal 14A] containing mAbs HIV-6, HIV-45 and HIV-125 to determine if this mixture of antibodies could recapitulate the 100% breadth of neutralization of the plasma against these pseudoviruses. Individually, mAbs HIV-6 and HIV-45 neutralized 75% of pseudoviruses tested, while HIV-125 neutralized only 17% of pseudoviruses tested, however the polyclonal mix 14A exhibited neutralizing activity against 100% of the pseudoviruses tested. Of the 12 pseudoviruses tested, the neutralizing activity exhibited by polyclonal mix 14A was characterized as strong (IC_50_ < 1 μg/mL) against 2 pseudoviruses, moderate (IC_50:_ 1–10 μg/mL) against 7 pseudoviruses, and weak (IC_50_ > 10 μg/mL) against 3 pseudoviruses. For subject VC20013, we tested pseudoviruses from 0.57 [five isolates], 1.01 [three isolates], 2.12 [one isolate], or 2.6 [three isolates], YPI. A total of 10 out of the 56 mAbs isolated from subject VC20013 exhibited neutralizing activity against at least one of the autologous pseudoviruses tested, with neutralization breadth ranging from 8 to 92% (Fig 3B). The differential binding and neutralization of all the mAbs generated from subject VC20013 is shown in (S3 Table). The genetic and functional features of these mAbs are summarized in (S5 Table). As some of these mAbs were isolated at 7 or 7.9 YPI, and the matching subject VC20013 plasma samples from the same time-points showed neutralizing activity when tested against 100% of this panel of autologous pseudoviruses, we tested a mixture [polyclonal 13A] containing mAbs HIV-733 and HIV-752 and observed 100% neutralizing activity against the panel of autologous pseudoviruses. Of the 12 pseudoviruses tested, the neutralizing activity exhibited by polyclonal mix 13A was characterized as strong (IC_50_ < 1 μg/mL) against 10 pseudoviruses, and moderate (IC_50:_ 1–10 μg/mL) against 2 pseudoviruses. These data suggest that polyclonal mixtures of mAbs that each mediates a limited breadth of neutralization, can cooperatively achieve higher levels of neutralization breadth against a panel of autologous viruses.

### Heterologous breadth of neutralization

**Fig 4:**
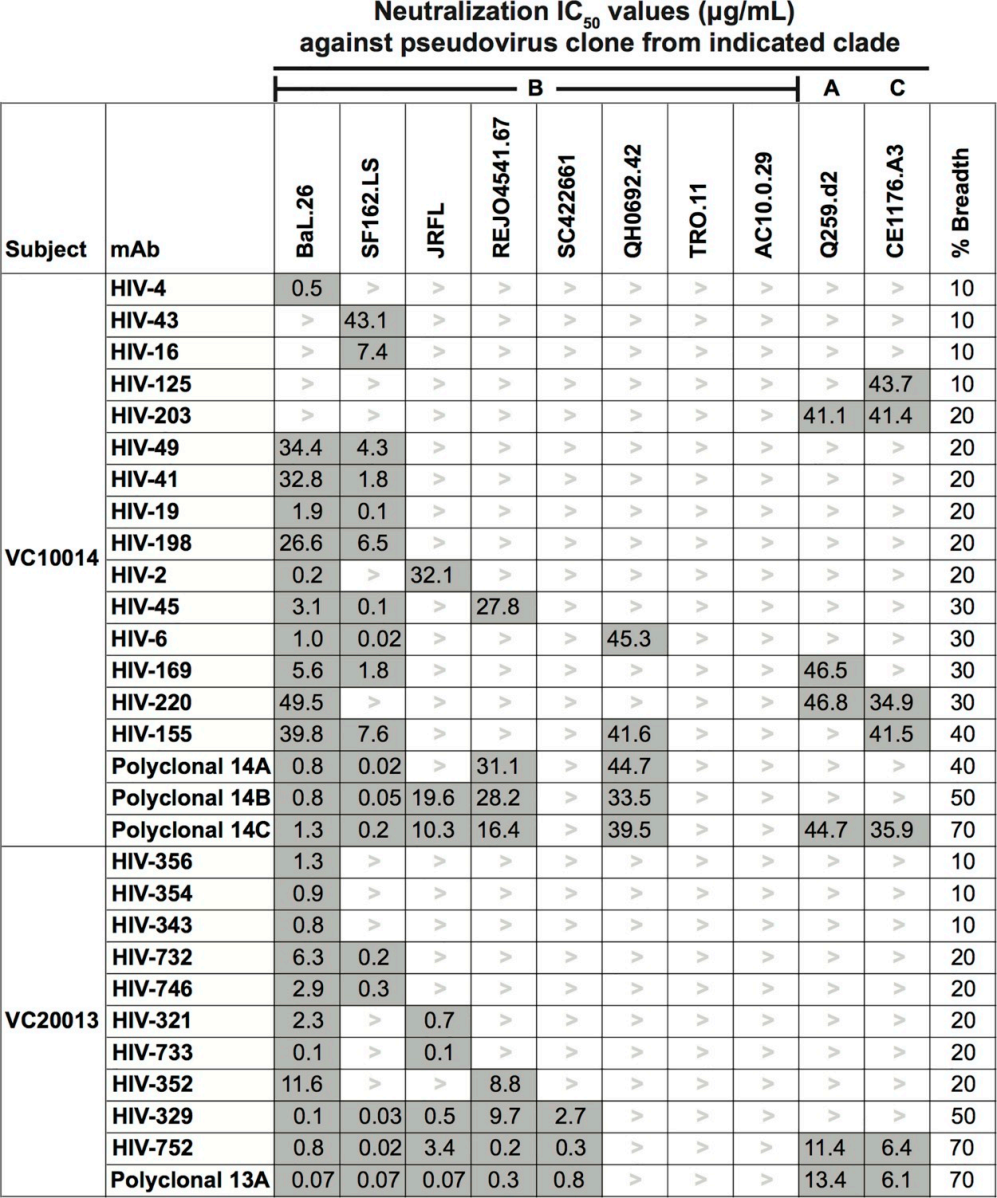
Heterologous neutralization breadth of HIV-1 mAbs. The neutralization phenotypes of HIV-1 mAbs and polyclonal mixtures from subjects VC10014 and VC20013 were determined using neutralization assays against heterologous pseudoviruses from the indicated clades. The values reported here shaded in grey are the IC_50_ values of neutralization as antibody concentration in μg/mL. The percent breadth reported is calculated by dividing the number of pseudoviruses for which an IC_50_ was recorded by the total number of pseudoviruses tested and multiplying by 100.

Since plasma samples from subjects VC10014 and VC20013 had been shown previously to develop serum-neutralizing antibodies against a broad spectrum of heterologous HIV-1 isolates, we sought to determine the heterologous neutralization breadth and potency of 15 mAbs from subject VC10014 and 10 mAbs from subject VC20013. As the subjects were infected in the U.S. with clade B viruses, we tested eight clade B strains that had been neutralized by plasma from both subjects. We also included one representative strain each for clades A and C. For subject VC10014, we detected strong neutralizing activity of individual mAbs against clade B isolates BaL.26 and SF162.LS, and moderate to weak neutralizing activity against strains JRFL, REJO4541.67 and QH0692.42 (Fig 4). Although plasma from subject VC10014 neutralized the Tier 2 clade B viruses SC422661, TRO.11, and AC10.0.29, we did not isolate any mAbs from this subject that individually neutralized these viruses. Several mAbs exhibited weak neutralizing activity against the clade A and C isolates tested, and interestingly, we identified two mAbs, HIV-125 and HIV-203, that did not neutralize any of the clade B isolates but exhibited weak neutralizing activity against the clade A isolate Q259.d2 (HIV-203) and the clade C isolate CE1176.A3 (Fig 4). To determine whether we could achieve increased breath using polyclonal mixtures of mAbs with limited individual neutralization breadth, we tested polyclonal mixtures of mAbs from subject VC10014 against the same panel of viruses. Polyclonal mix 14A [mAbs HIV-6 + HIV-45 + HIV-125] neutralized only 40% of viruses tested, Polyclonal mix 14B [mAbs HIV-2 +HIV-6 + HIV-45] neutralized 50% of viruses tested, and polyclonal mix 14C [mAbs HIV-45 + HIV-155 + HIV-169] neutralized 70% of the viruses tested (Fig 4). For subject VC20013, we detected strong neutralizing activity against the clade B isolates BaL.26, SF162.LS, JRFL, REJO4541.67, and SC422661 for some mAbs, with other mAbs exhibiting moderate to weak neutralizing activity against these viruses. We did not detect any neutralizing activity by any mAb against QH0692.42, TRO.11, or AC10.0.29. The neutralization breadth of mAbs from subject VC20013 ranged from 10 to 70%. Although polyclonal mix 13A [mAbs HIV-733 + HIV-752] neutralized 70% of the viruses tested, which was comparable to the neutralization breadth observed for mAb HIV-752 (neutralized 70% of viruses tested), there was a marked increase in potency against two of the clade B viruses tested (Fig 4). Analysis of the genetic features of these NAbs to revealed that they did not have the genetic features associated with bNAbs which include very long HCDR3 regions, and very high somatic mutation percentages in the heavy chain variable region (S5 Table), indicating that these mAbs did not possess similar genetic features as some of the canonical bNAbs.

### Epitope mapping HIV-1 mAbs

To better characterize the antigenic landscape of the panel of mAbs isolated from both subjects, we performed epitope mapping using binding of mAbs to a collection of 15-mer overlapping peptides based on the HIV-1 group M consensus sequence of Env. We identified the epitope for a number of mAbs from both subjects that showed binding to linear peptides across the different regions of the HIV-1 Env (Panel A in S1 Fig), for subject VC10014; and Panel B in S1 Fig, for subject VC20013.) We did not detect binding to any peptides for 80% or 86% of the mAbs isolated from subjects VC10014 or VC20013 respectively, suggesting that those mAbs target conformationally complex epitopes on the HIV-1 Env. We next sought to classify the mAbs into different groups in order to determine the representation of different epitope specificities within the antibody repertoire of both subjects. Using a classical sandwich epitope binning format where mAbs of similar epitope specificities showed similar binning phenotypes, we identified several mAbs that exhibited a competitive relationship, and clustered together into groups (Panel A in S2 Fig). The relationship of each mAb to other mAbs tested is shown in a bin network plot combining mAbs from both subjects (S6 Table) shows the mAbs from each subject used in the bin network plot, with shared colors indicating bins of mAbs with identical competitive relationships with other mAbs in the assay, lines representing a competitive relationship between mAbs, and dashed lines indicating asymmetric competition (Panel B S2 Fig). Through analysis of binding patterns observed for several mAbs to HIV-1 Env mutants, we were able to determine that group 1 and 2 mAbs were CD4bs-specific, and group 3 mAbs are V3-specific (S7 Table). The findings here revealed that several of the mAbs isolated from both subjects target the same antigenic regions on the HIV-1 Env.

### Epitope specificity of neutralizing antibodies

**Fig 5:**
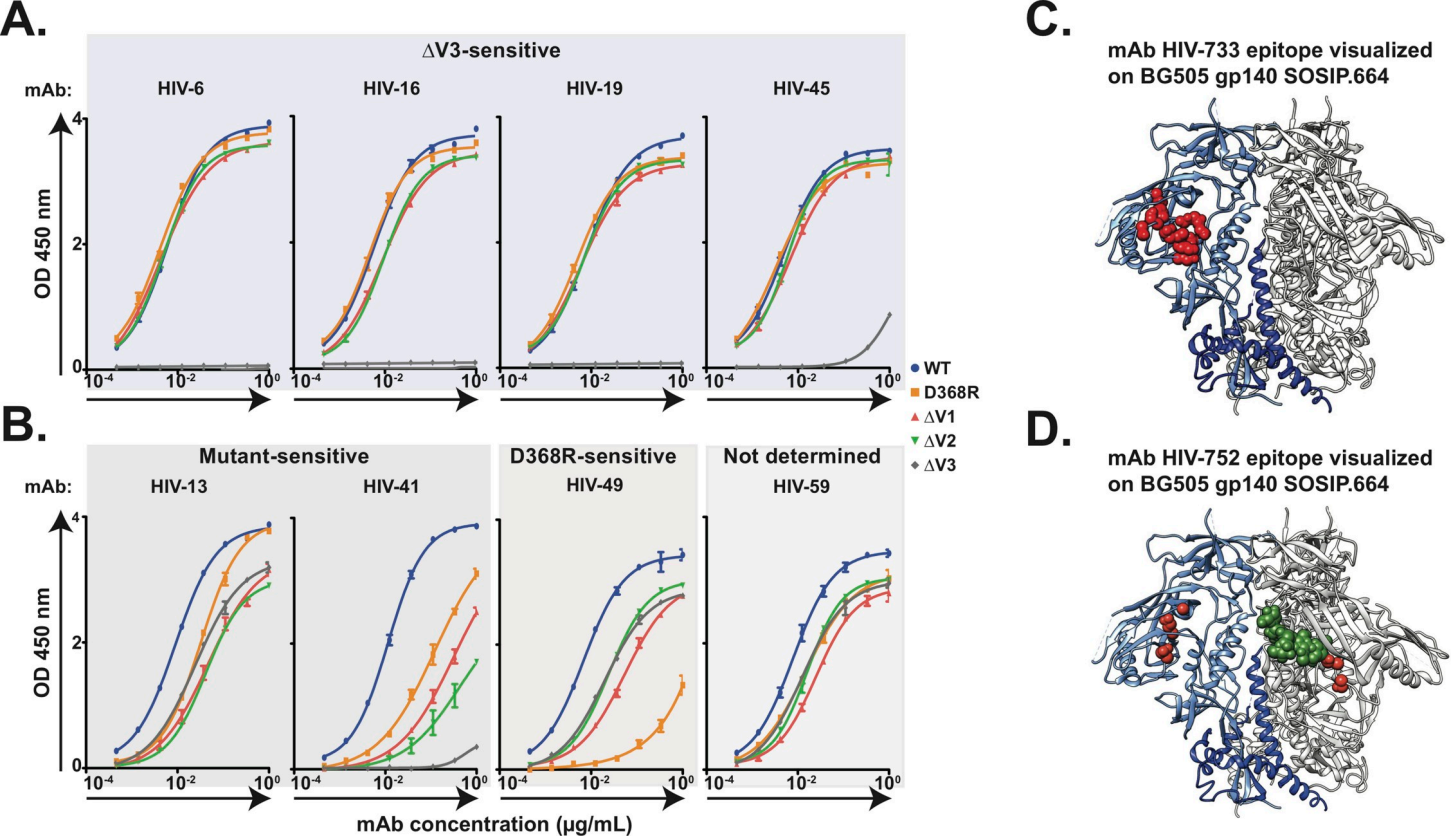
Epitope mapping neutralizing HIV-1 mAbs from subjects VC10014 and VC20013. ELISA was used to determine the epitopes of several HIV-1 NAbs by testing binding to SF162 *wt* Env, SF162 ΔV1, SF162 ΔV2, SF162 ΔV3, and SF162 D368R mutants. **(A)** Binding phenotype of V3-specific mAbs determined by ELISA. **(B)** Binding phenotype of CD4-specific mAbs determined by ELISA. Using a comprehensive library of HIV-1 Env mutants, cytometric flow sorting was used to identify critical residues for the binding of HIV-1 mAbs. **(C)** Visualization of mAb HIV-733 epitope (indicated in red) on the crystal structure of BG505 gp140 SOSIP.664. **(D)** Visualization of mAb HIV-752 epitope (indicated in red) and critical residues at the N262 glycosylation site (indicated in green) on the crystal structure of BG505 gp140 SOSIP.664.

As the neutralizing mAbs from both subjects displayed diverse neutralization patterns against the panel of pseudoviruses used, we sought to determine the antigenic regions targeted by these mAbs on the SF162 HIV-1 Env using variable loop deletion and D368R Env mutants (S7 Table) since the neutralizing epitope in serum from subject VC10014 had been mapped to the CD4bs. We identified a group of NAbs that showed binding to the *wt* SF162 Env and mutant Envs tested, except the V3-deletion mutant, which resulted in complete loss of binding. This finding indicated that these NAbs targeted the V3 loop region of the HIV-1 Env (Fig 5A). Interestingly, these V3-specific NAbs had diverse neutralization patterns against autologous and heterologous viral isolates (Fig 3A and Fig 4), indicating that small variations in the variable regions can impact the ability of a NAb to neutralize specific viruses. In addition, we identified a group of NAbs that showed reduced binding to the mutant Envs compared to *wt* Envs. MAbs HIV-13 and HIV-41 showed reduced binding to all the mutants tested, and competition experiments revealed that they were grouped in the same competition-binding group with mAb HIV-49, which showed reduced binding to all the mutants, but was most sensitive to the D368R mutant, suggesting that these NAbs could be binding in a region near the bNAb b12 epitope in the CD4bs of the HIV-1 Env (Fig 5B). We also tested mAb HIV-59 that showed very strong binding to all the autologous Envs but did not neutralize any of the matching pseudoviruses (S4 Table). Competition-binding studies revealed that mAb HIV-59 did not compete with the other CD4bs NAbs, indicating that it could be targeting another region of the HIV-1 Env. Using cell-based binding assays against a comprehensive library of HIV-1 Env mutants with alanine substituted into each position on the gp160 of the KNH1144 strain, we determined the critical residues for mAb HIV-733 and mAb HIV-752 recognition of Env (S8 Table). Analysis of the critical residues revealed that mAb HIV-733 targets a site in or near the CD4 binding site on HIV-1 (Fig 5C), and mAb HIV-752 also targets an epitope in the CD4bs in addition to an asparagine at position 262 (HXB2 numbering) which is a glycosylation site on the HIV-1 Env (Fig 5D). These findings indicate that the neutralizing activity observed in both subjects is mediated by NAbs that individually target distinct epitopes, specifically the CD4bs and V3 loop of HIV-1.

## Discussion

The mechanism driving co-evolution of the polyclonal antibody repertoire and HIV-1 quasispecies diversification during the emergence of broadly neutralizing responses in HIV-1-infected subjects has not been fully elucidated. Studies investigating the co-evolution of a founder virus and a broadly neutralizing antibody revealed that viral selection and epitope diversification in the founder virus lineage preceded the development of broadly neutralizing activity [34], consistent with other studies that have highlighted viral diversification as an important mediator of developing neutralization breadth [35]. In subjects VC10014 and VC20013 studied here, the appearance of breadth in the antibody repertoire coincided with the diversification of the circulating autologous Envs, indicating that increased viral diversity in these subjects likely drove the development of increased neutralizing breadth observed in their antibody repertoires [38].

The neutralization breadth observed in previous studies using plasma from subjects VC10014 and VC20013 was mapped earlier in infection mostly to particular dominant specificities in the Env protein [11,38]. For subject VC10014, this serological breadth was mapped to an epitope overlapping the CD4 binding site, while the serological breadth of subject VC20013 was mapped to the MPER region of gp41 [38]. Broadly neutralizing antibody responses targeting specific epitopes have been reported to account for the majority of the broadly neutralizing responses against heterologous isolates in some subjects [50]. In other subjects, it has been reported that coverage of multiple epitope targets could be driving the observed breadth [51]. The data here from studies with mAbs isolated from chronically infected subjects VC10014 and VC20013 indicates that a significant level of autologous breadth using a polyclonal combination of mAbs targeting multiple viral epitopes in these subjects could be achieved, even though no single mAb exhibited the breadth of binding and neutralization seen in the subjects’ plasma samples. The biased recognition of certain Env antigenic sites seen in some plasma samples from these subjects was not reflected in the restriction of antigen recognition observed in the panel of mAb clones we obtained. It is important to note that for subject VC20013, serological breadth was mapped to the MPER region using plasma from 2.60 YPI and earlier, while the neutralizing mAbs were isolated from memory B cells collected at 7.9 YPI. It is possible that a shift from MPER to CD4 binding site as the predominant epitope driving breadth could have occurred between 2.6 and 7.9 YPI in this subject, consistent with other studies showing sequential targeting of different Env epitopes with elicitation of different breadth profiles [52]. One limitation of our studies is that we performed the studies prior to development of native-like trimers [53,54,55]; the more open trimeric Env constructs we used likely were not able to identify any rare bNAbs present that only bind to closed native-like trimers.

Our studies using supernatants from EBV-transformed B cells and purified mAbs isolated from subjects VC10014 and VC20013 revealed the presence of antibodies that differentially recognized autologous gp140 proteins and pseudoviruses. Interestingly, we observed that some of the mAb binding and neutralization patterns were restricted to specific time-points. For subject VC20013, from whom multiple viral variants were isolated at the same time-point, we observed mAbs that showed specificity to some viral isolates but not to other viruses from the exact same time-point and plasma sample. It is well established that following HIV-1 infection the initial autologous neutralizing antibody response drives virus escape, and as a result, the antibody repertoire constantly chases evolving virus variants. This phenomenon could lead to restricted recognition of certain specificities in the neutralizing antibody repertoire of most HIV-1-infected subjects. In subjects VC10014 and VC20013, differential viral recognition by individual mAbs indicated that particular antibody lineages might have been focused on specific viral isolates, and the eventual breadth of these individual mAbs could have been determined by the particular virus target of the earliest mAb response. There is evidence that certain Envs induce more breadth and potency than others; for example, studies have shown that rhesus macaques infected with the CCR5-tropic SHIV-AD8 virus often develop neutralization breadth [56]. These findings indicate that the initial recognition of a particular viral isolate by an antibody can result in immunological focusing, thereby restricting the potential of that antibody clonal family to acquire breadth. In a separate study, specific Envs from these subjects have been used as vaccines and have shown that they can elicit potent autologous neutralizing antibodies and modest Tier 2 heterologous neutralization [57].

One of the more interesting findings from this study was that the differential pattern of autologous versus heterologous neutralization observed for certain mAbs isolated from subject VC20013. MAb HIV-733 isolated from 7.9 YPI neutralized 92% of the autologous viruses tested but neutralized only 20% of the heterologous viruses tested. Remarkably, although mAb HIV-329 isolated at 2.60 YPI neutralized only 20% of the autologous viruses tested, it neutralized 50% of the heterologous viruses tested (Fig 3B and Fig 4). It is important to note that all the autologous viruses tested were from 2.60 YPI and earlier, raising the possibility that compared to mAb HIV-329 isolated at the same time point, mAb HIV-733 isolated from a later time point probably was selected against by fewer autologous escape mutants, hence the impression that it has higher breadth than mAb HIV-329. The neutralization findings from mAb HIV-733 suggest that the development of autologous neutralization depth does not necessarily correlate strictly with the acquisition of neutralization breadth for heterologous Envs. The observed difference in autologous neutralization depth and heterologous neutralization breadth between mAbs HIV-329 and HIV-733 likely was driven by particular residues in the epitopes targeted. MAb HIV-329 targets an epitope in the V3 region of HIV-1 Env, and it is likely that within the autologous quasispecies, the mAb HIV-329 epitope in this highly variable region continuously diversifies as escape mutants emerge. This quasispecies diversification resulted in limited neutralization depth, as is evidenced by the fact that mAb HIV-329 neutralized only 20% of the autologous isolates tested. MAb HIV-733, however, targets an epitope in the CD4bs, which likely was more conserved in the autologous quasispecies, resulting in mAb HIV-733 neutralizing 92% of autologous viruses tested.

Previous studies have shown that a combination of bNAbs targeting multiple epitopes can increase neutralization breadth [58]. Our study reveals insights into the neutralization landscape covered by a panel of mAbs with differential specificities within HIV-1 infected subjects. As specific antibodies target individual viruses within the quasispecies, an aggregation of these specificities can result in the development of neutralization breadth against autologous viral isolates. This alternate polyclonal mechanism stands in contrast to current paradigms where the focus has been on finding the rare single autologous bNAb that can individually exhibit breath of neutralization against heterologous viral isolates. An alternate mechanism for achieving breadth could be through the induction of populations of antibodies with limited breadth individually, that can function in concert to mediate neutralization breadth against diverse viruses.

## Supporting information

S1 Fig Linear peptide mapping of HIV-1 mAbs. ELISA was used to determine the number of mAbs from each donor recognizing overlapping linear peptides of the HIV-1 Env. **(A)** Number of mAbs from subject VC10014 recognizing overlapping linear peptides, and envelope regions where peptides are located. **(B)** Number of mAbs from subject VC20013 recognizing overlapping linear peptides, and envelope regions where peptides are located. The height of each line represents the number of mAbs recognizing that peptide. Env regions recognized by HIV-1-specific mAbs from both subjects are indicated in orange color.
(TIFF)

S2 Fig Competition grouping of HIV-1 mAbs. Epitope binning was used to group mAbs from subject VC10014 into different competition groups. **(A)** Grouping of mAbs from subject VC10014 into different competition groups. Reductions of >70% in maximal signal of the competing mAb is shaded in black. Reductions of <30% in maximal signal of the competing mAb is shaded in white. Intermediate competition defined as between 30% - 70% reduction in maximal signal of the competing mAb is shaded in grey. **(B)** Network plot showing groupings and associations of mAbs from subjects VC10014 and VC20013. Each color-coded group indicates mAbs with a competitive relationship. Lines represent blocking relationships in the network, and dashed lines represent asymmetric competition. Group 1 mAbs (blue) and group 2 mAbs (green) are CD4bs-specific. Group 3 mAbs (orange) are V3-specific.
(TIFF)

S1 Table Summary of hybridoma generation from HIV-1 infected subjects. (TIFF)

S2 Table List of autologous Envs from subjects VC10014 and VC20013. (TIFF)

S3 Table Differential binding and neutralization of subject VC20013 mAbs. (TIFF)

S4 Table Differential binding and neutralization of subject VC10014 mAbs. (TIFF)

S5 Table Genetic and functional features of mAbs isolated from subjects VC10014 and VC20013. (TIFF)

S6 Table MAbs from subjects VC10014 and VC20013 used to generate binning network plot. (TIFF)

S7 Table Heatmap of HIV-1 mAbs binding to SF162 gp140 mutants. (TIFF)

S8 Table Summary of gp160 residues critical for mAbs HIV-733 and HIV-752 binding. (TIFF)
