# Supplementary figures and images for: Increased breadth of HIV-1 neutralization achieved by diverse antibody clones each with limited neutralization breadth
Source: PLoS One. 2018 Dec 19;13(12):e0209437. doi: 10.1371/journal.pone.0209437 (PMC6300260; doi:10.1371/journal.pone.0209437)

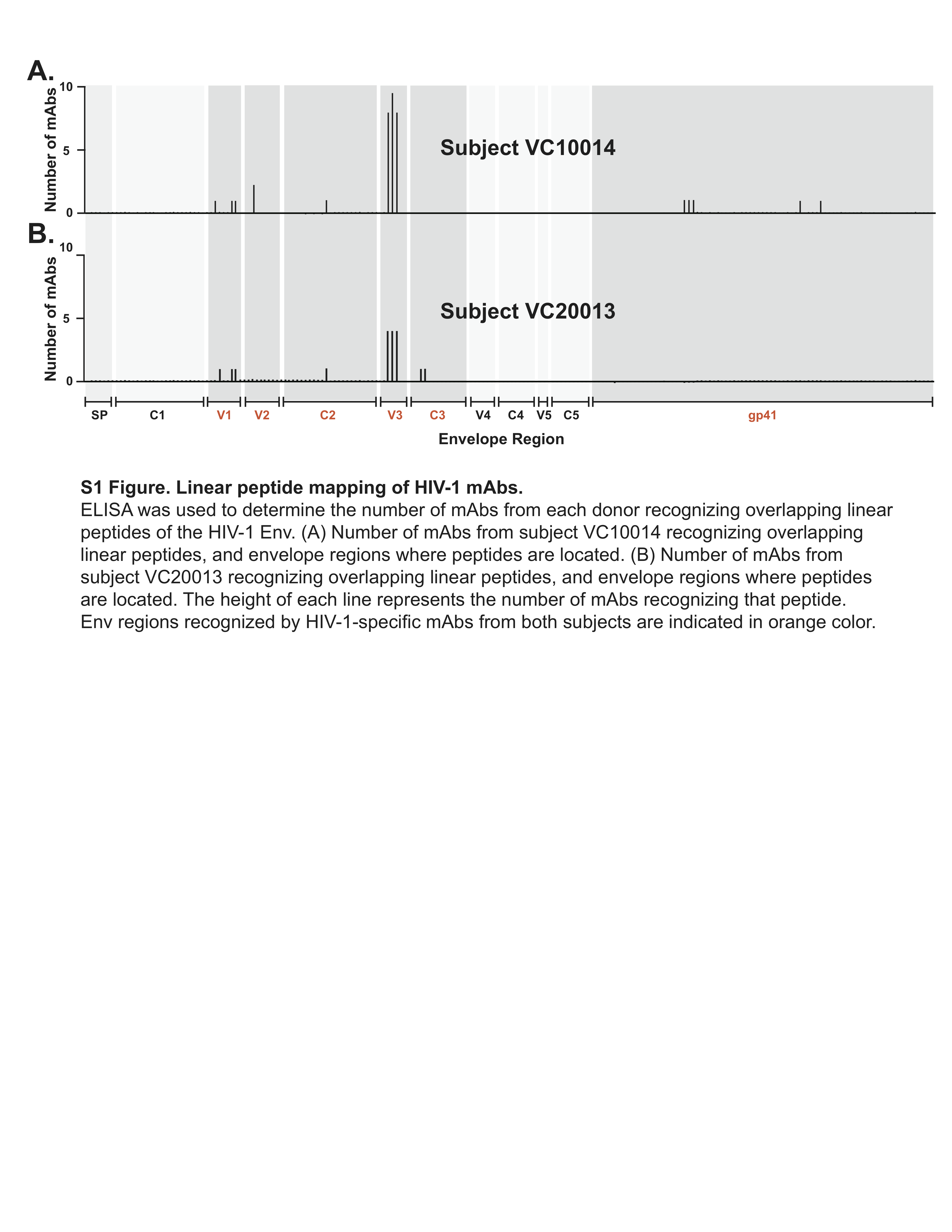

Supplement: S1 Fig — ELISA was used to determine the number of mAbs from each donor recognizing overlapping linear peptides of the HIV-1 Env. (A) Number of mAbs from subject VC10014 recognizing overlapping linear peptides, and envelope regions where peptides are located. (B) Number of mAbs from subject VC20013 recognizing overlapping linear peptides, and envelope regions where peptides are located. The height of each line represents the number of mAbs recognizing that peptide. Env regions recognized by HIV-1-specific mAbs from both subjects are indicated in orange color. (TIFF) [file pone.0209437.s001.tiff]

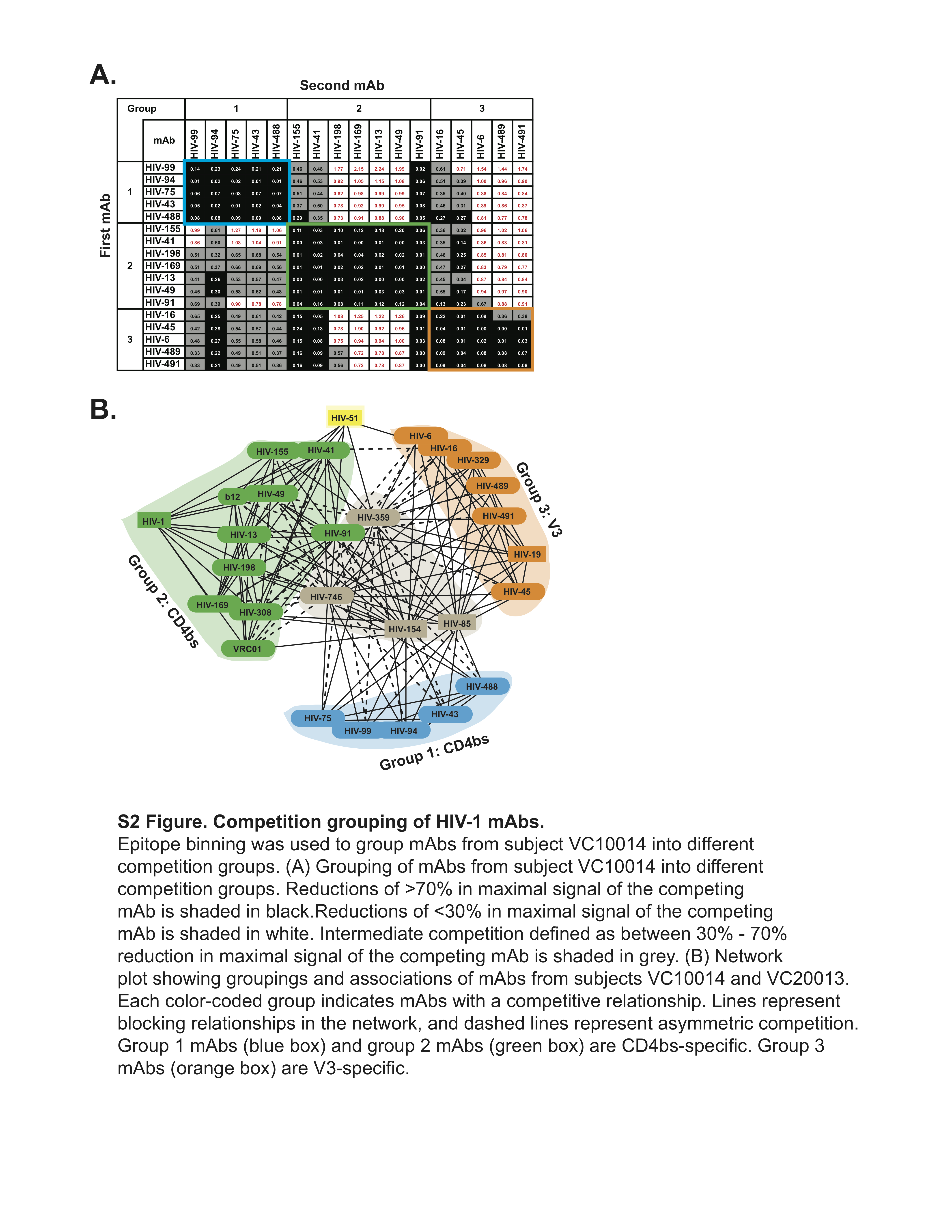

Supplement: S2 Fig — Epitope binning was used to group mAbs from subject VC10014 into different competition groups. (A) Grouping of mAbs from subject VC10014 into different competition groups. Reductions of >70% in maximal signal of the competing mAb is shaded in black. Reductions of <30% in maximal signal of the competing mAb is shaded in white. Intermediate competition defined as between 30% - 70% reduction in maximal signal of the competing mAb is shaded in grey. (B) Network plot showing groupings and associations of mAbs from subjects VC10014 and VC20013. Each color-coded group indicates mAbs with a competitive relationship. Lines represent blocking relationships in the network, and dashed lines represent asymmetric competition. Group 1 mAbs (blue) and group 2 mAbs (green) are CD4bs-specific. Group 3 mAbs (orange) are V3-specific. (TIFF) [file pone.0209437.s002.tiff]

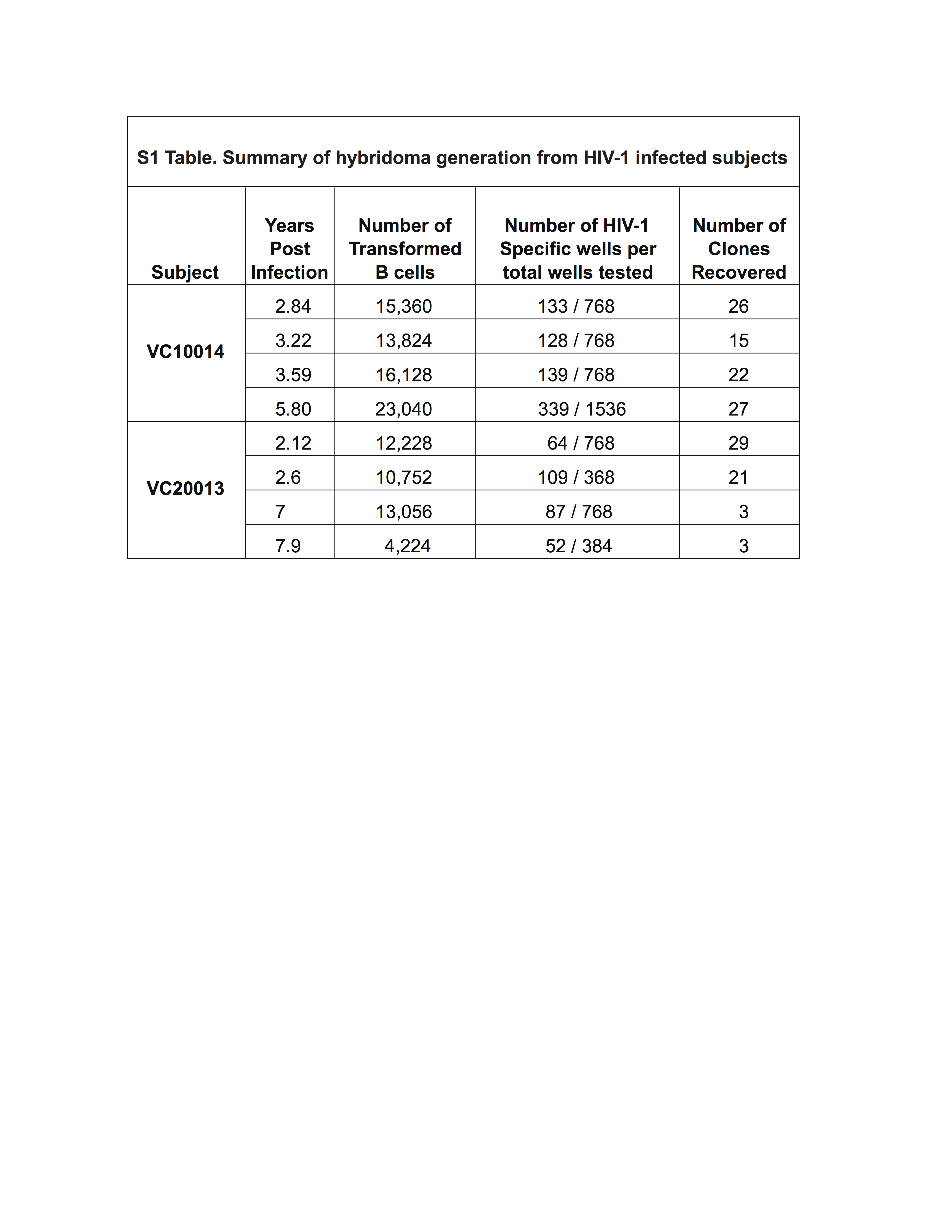

Supplement: S1 Table — (TIFF) [file pone.0209437.s003.tiff]

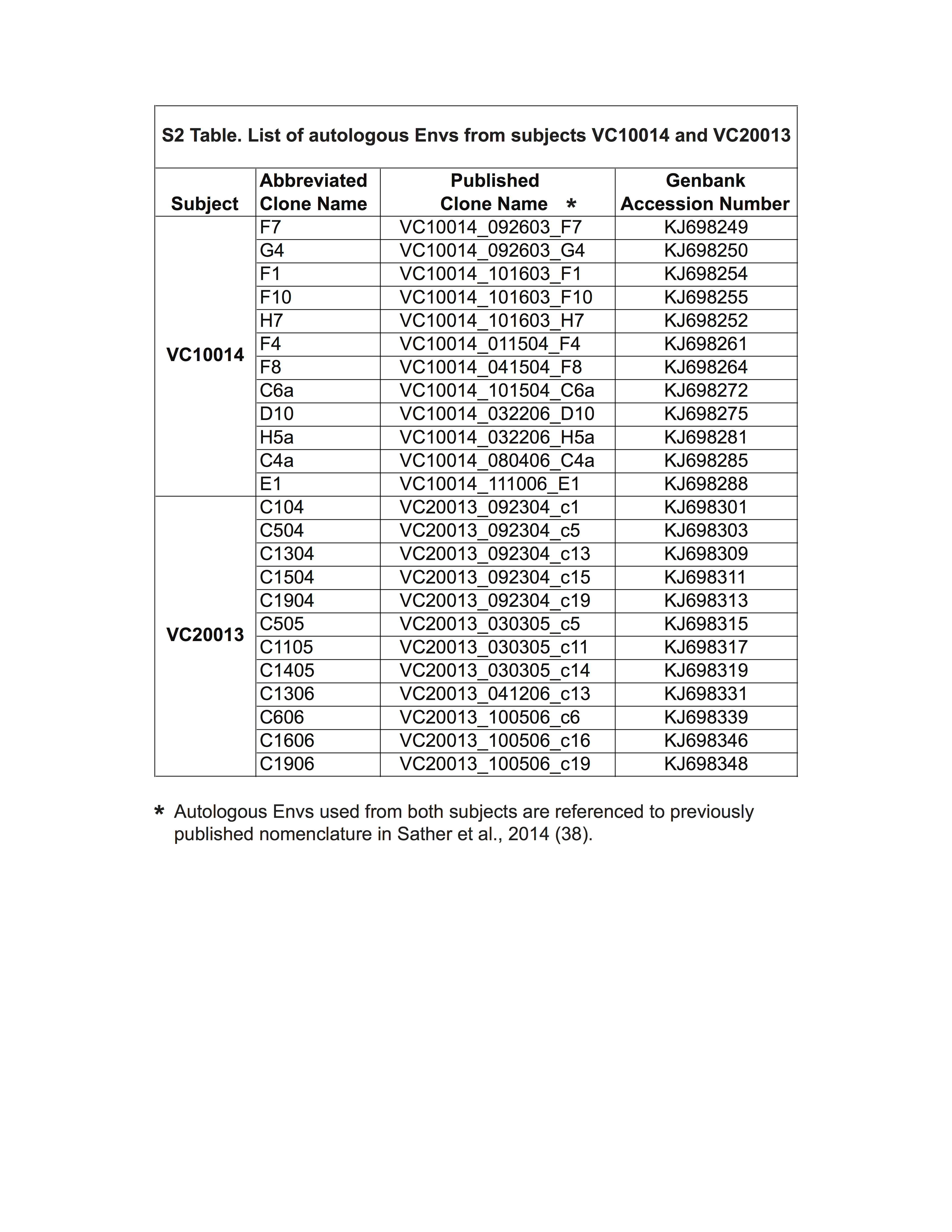

Supplement: S2 Table — (TIFF) [file pone.0209437.s004.tiff]

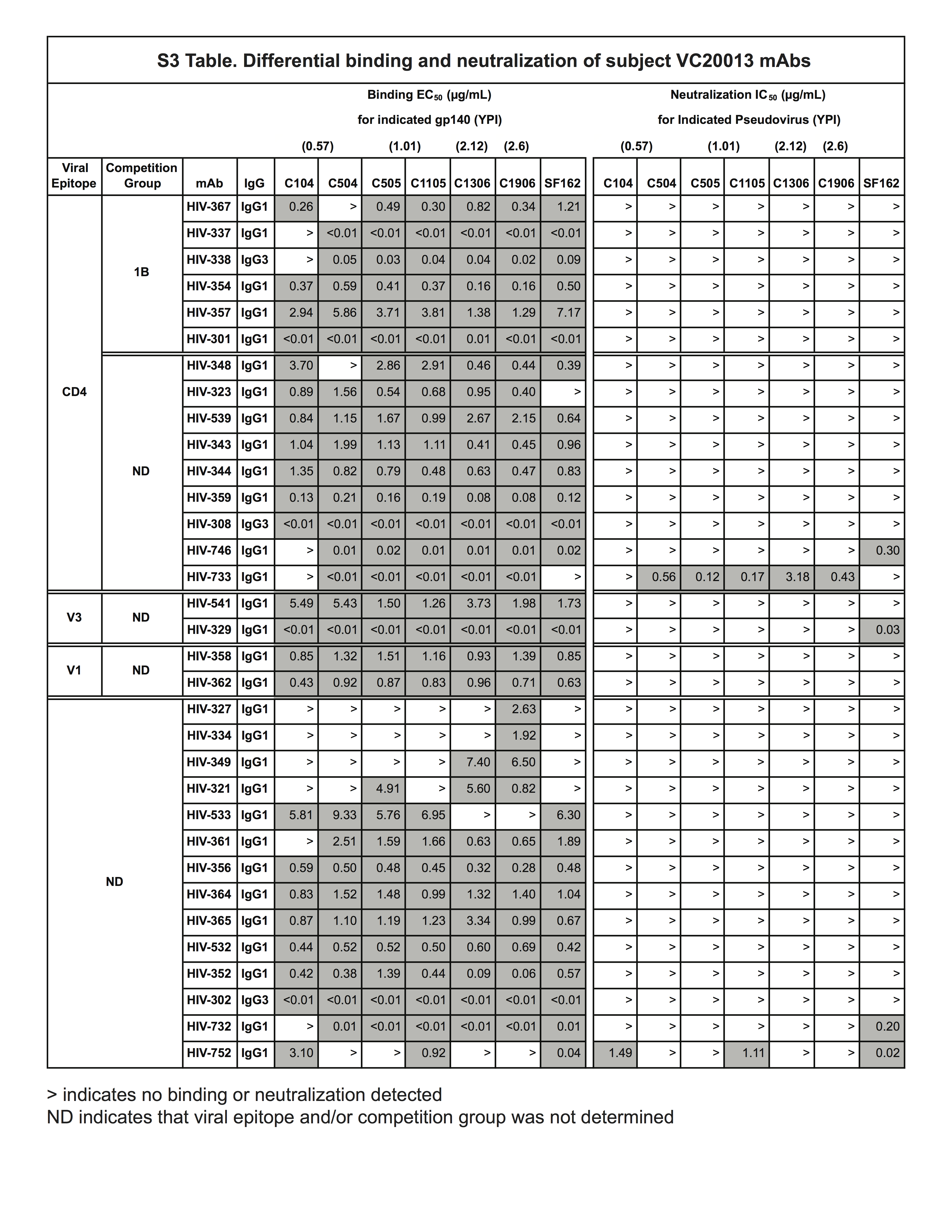

Supplement: S3 Table — (TIFF) [file pone.0209437.s005.tiff]

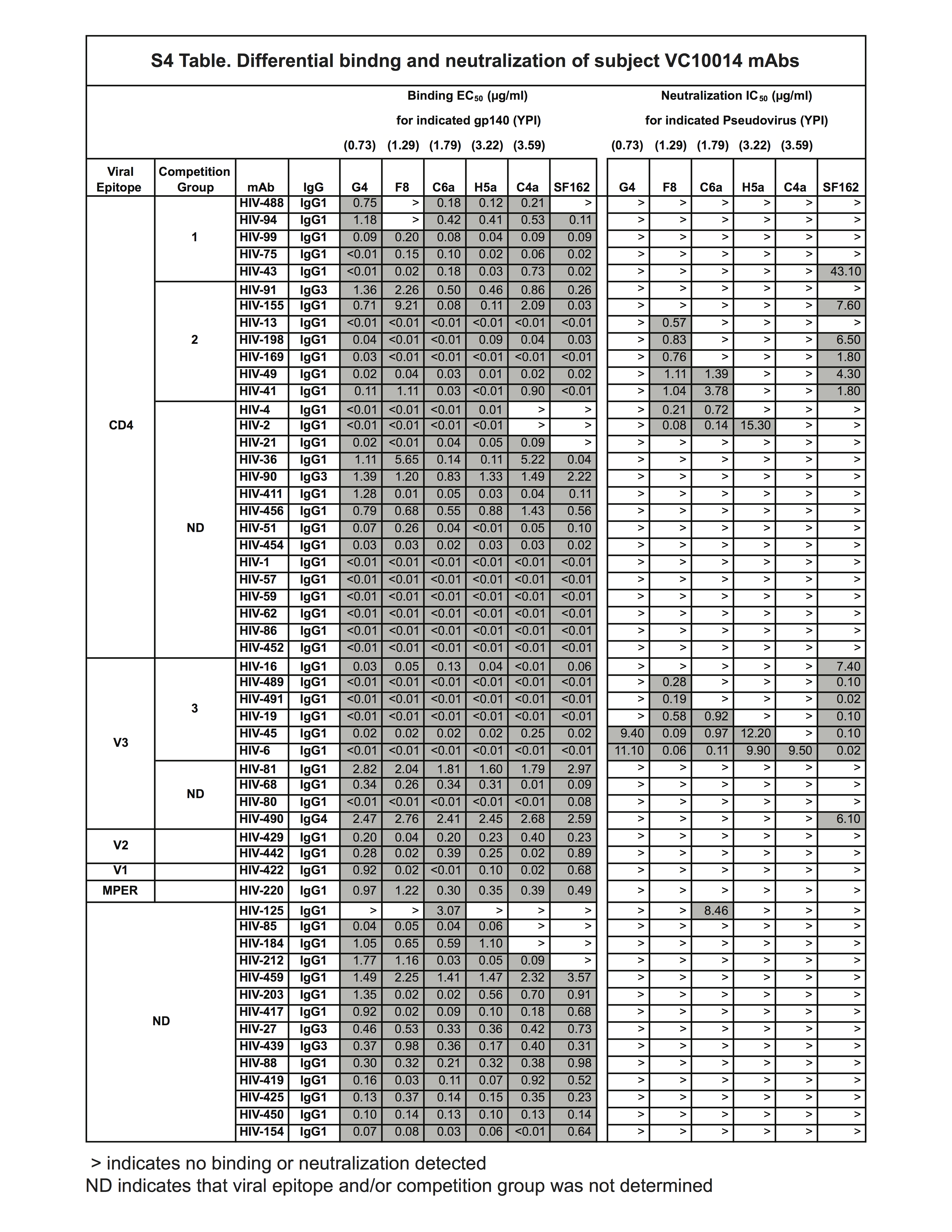

Supplement: S4 Table — (TIFF) [file pone.0209437.s006.tiff]

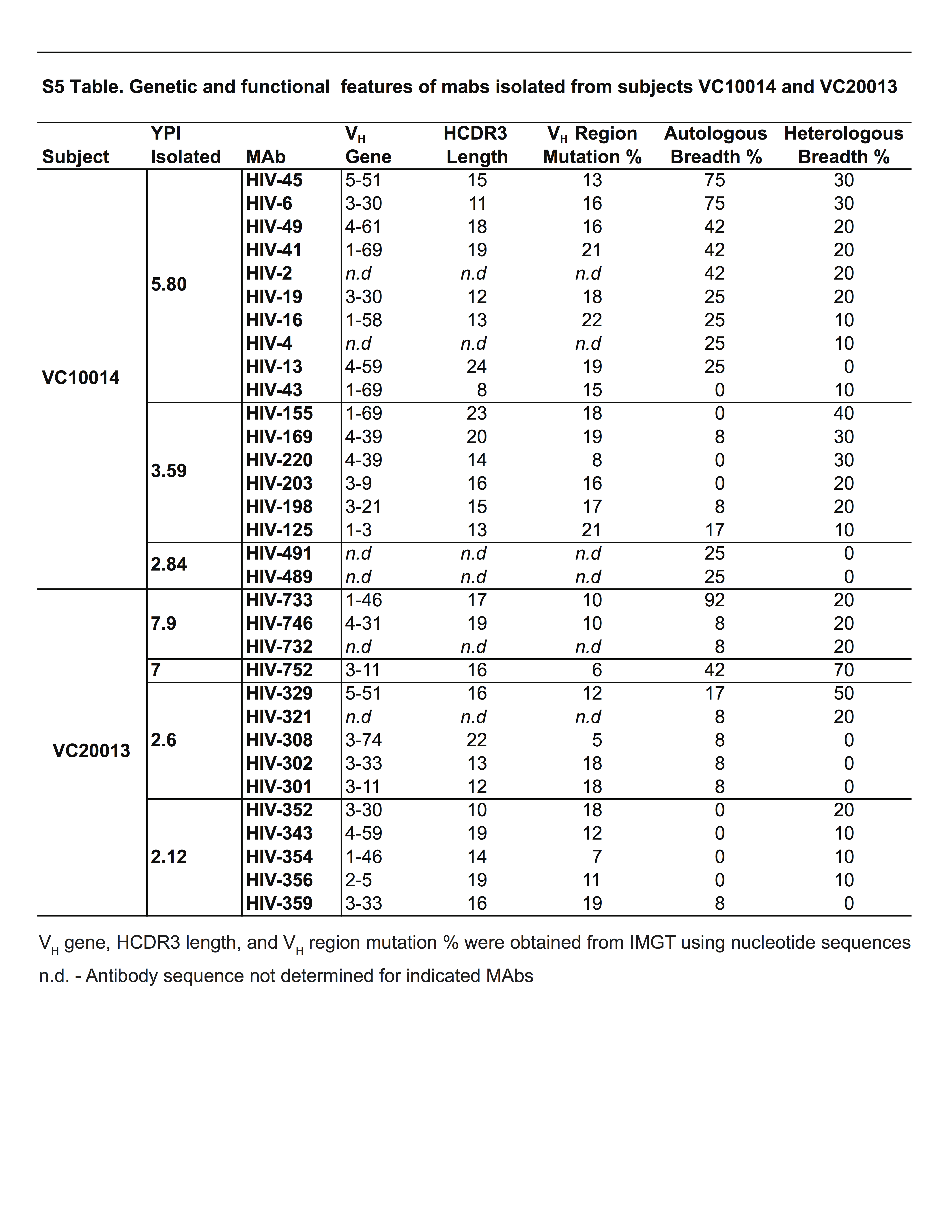

Supplement: S5 Table — (TIFF) [file pone.0209437.s007.tiff]

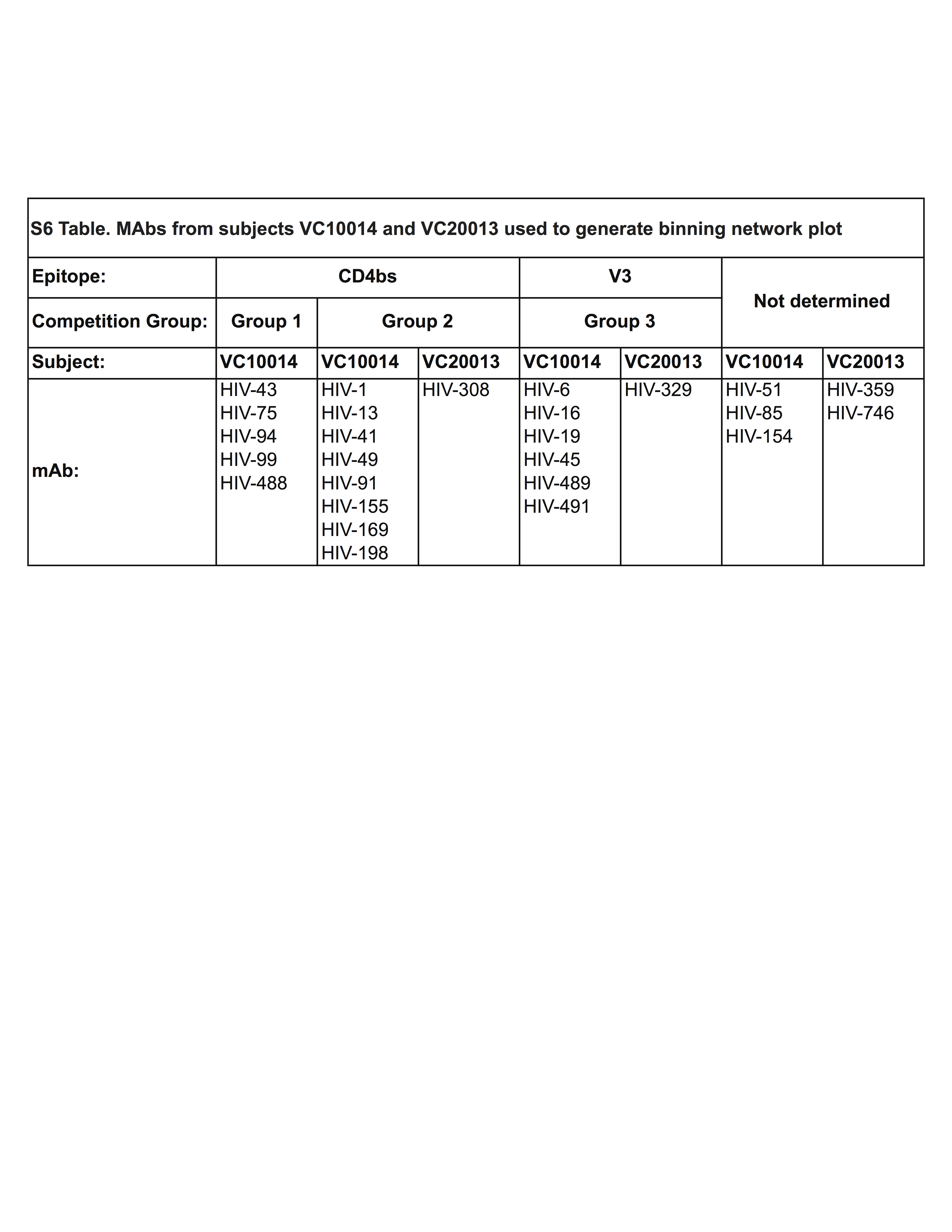

Supplement: S6 Table — (TIFF) [file pone.0209437.s008.tiff]

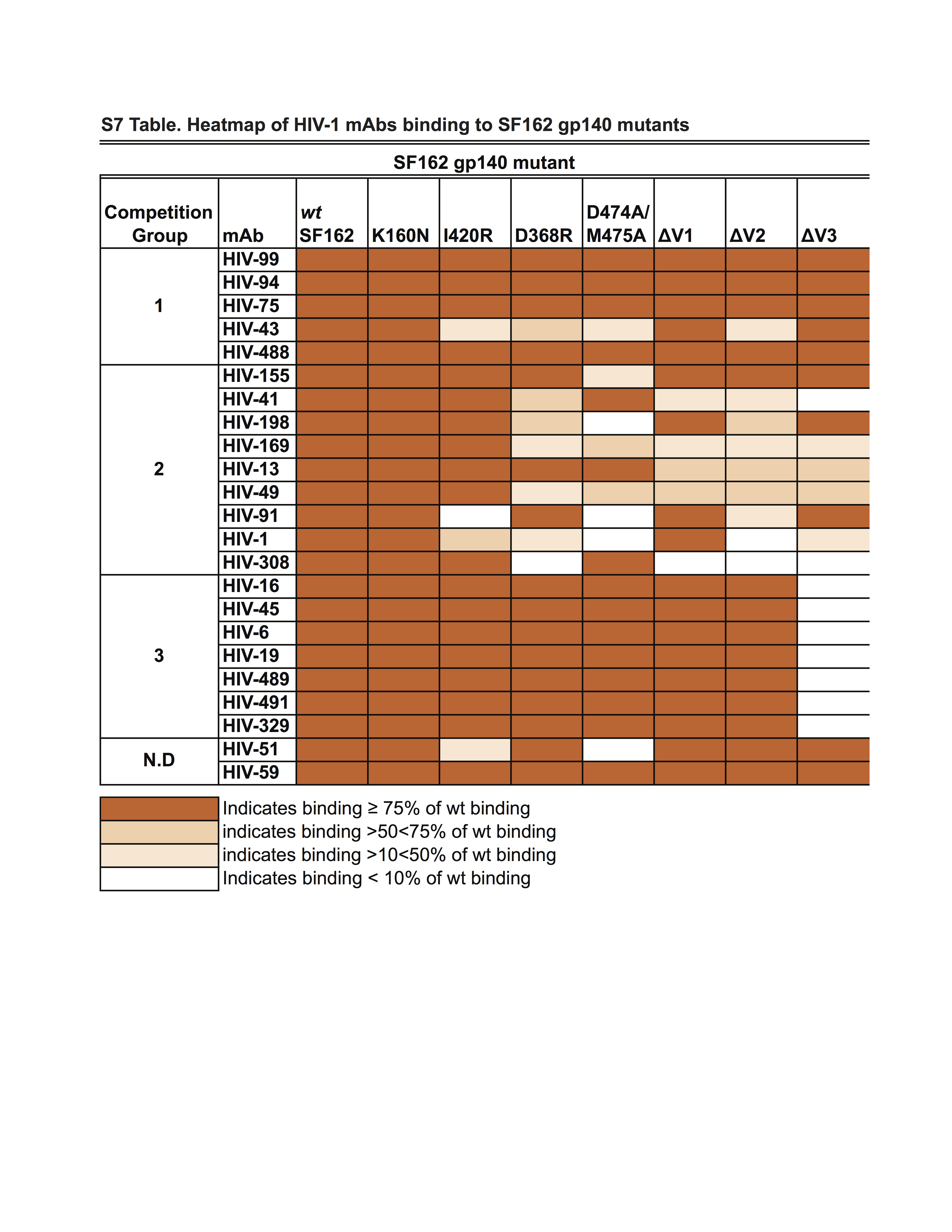

Supplement: S7 Table — (TIFF) [file pone.0209437.s009.tiff]

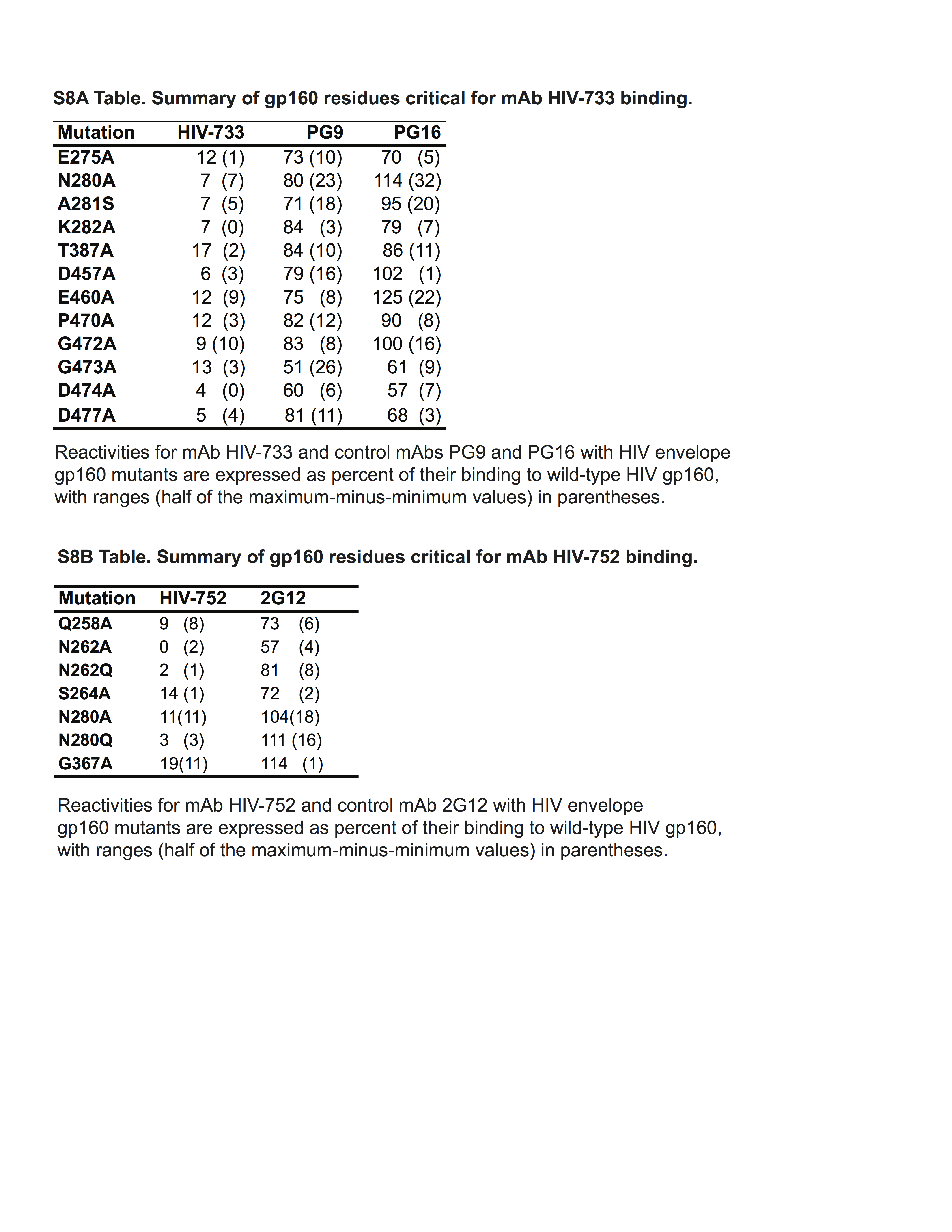

Supplement: S8 Table — (TIFF) [file pone.0209437.s010.tiff]
